# A novel Menin-MLL1 inhibitor, DS-1594a, prevents the progression of acute leukemia with rearranged *MLL1* or mutated *NPM1*

**DOI:** 10.1186/s12935-023-02877-y

**Published:** 2023-02-25

**Authors:** Masashi Numata, Noriyasu Haginoya, Machiko Shiroishi, Tsuyoshi Hirata, Aiko Sato-Otsubo, Kenji Yoshikawa, Yoshimi Takata, Reina Nagase, Yoshinori Kashimoto, Makoto Suzuki, Nina Schulte, Gernot Polier, Akiko Kurimoto, Yumiko Tomoe, Akiko Toyota, Tomoko Yoneyama, Emi Imai, Kenji Watanabe, Tomoaki Hamada, Ryutaro Kanada, Jun Watanabe, Yoshiko Kagoshima, Eri Tokumaru, Kenji Murata, Takayuki Baba, Taeko Shinozaki, Masami Ohtsuka, Koichi Goto, Tsuyoshi Karibe, Takao Deguchi, Yoshihiro Gocho, Masanori Yoshida, Daisuke Tomizawa, Motohiro Kato, Shinji Tsutsumi, Mayumi Kitagawa, Yuki Abe

**Affiliations:** 1 Shinagawa R&D Center, Daiichi Sankyo Co., Ltd, 1-2-5 Hiromachi, Shinagawa-Ku, Tokyo, 140-0005 Japan; 2 Daiichi Sankyo RD Novare Co., Ltd, Tokyo, Japan; 3 Department of Pediatric Hematology and Oncology Research, National Research Institute for Child Health and Development, Tokyo, Japan; 4 Department of Pediatrics, University of Tokyo, Tokyo, Japan; 5 Daiichi Sankyo Europe GmbH, Munich, Germany; 6 Children’s Cancer Center, National Center for Child Health and Development, Tokyo, Japan

**Keywords:** Menin-MLL1 inhibitor, *MLL1-r* or *NPM1c* acute leukemia, Leukemia-initiating cells

## Abstract

**Background:**

*Mixed lineage leukemia 1-rearranged* (*MLL1-r*) acute leukemia patients respond poorly to currently available treatments and there is a need to develop more effective therapies directly disrupting the Menin‒MLL1 complex. Small-molecule–mediated inhibition of the protein‒protein interaction between Menin and MLL1 fusion proteins is a potential therapeutic strategy for patients with *MLL1-r* or mutated-*nucleophosmin 1* (*NPM1c*) acute leukemia. In this study, we preclinically evaluated the new compound DS-1594a and its salts.

**Methods:**

We evaluated the preclinical efficacy of DS-1594a as well as DS-1594a·HCl (the HCl salt of DS-1594a) and DS-1594a·succinate (the succinic acid salt of DS-1594a, DS-1594b) in vitro and in vivo using acute myeloid leukemia (AML)/acute lymphoblastic leukemia (ALL) models.

**Results:**

Our results showed that *MLL1-r* or *NPM1c* human leukemic cell lines were selectively and highly sensitive to DS-1594a·HCl, with 50% growth inhibition values < 30 nM. Compared with cytrabine, the standard chemotherapy drug as AML therapy, both DS-1594a·HCl and DS-1594a·succinate mediated the eradication of potential leukemia-initiating cells by enhancing differentiation and reducing serial colony-forming potential in *MLL1-r* AML cells in vitro. The results were confirmed by flow cytometry, RNA sequencing, RT‒qPCR and chromatin immunoprecipitation sequencing analyses. DS-1594a·HCl and DS-1594a·succinate exhibited significant antitumor efficacy and survival benefit in MOLM-13 cell and patient-derived xenograft models of *MLL1-r* or *NPM1c* acute leukemia in vivo.

**Conclusion:**

We have generated a novel, potent, orally available small-molecule inhibitor of the Menin-MLL1 interaction, DS-1594a. Our results suggest that DS-1594a has medicinal properties distinct from those of cytarabine and that DS-1594a has the potential to be a new anticancer therapy and support oral dosing regimen for clinical studies (NCT04752163).

**Supplementary Information:**

The online version contains supplementary material available at 10.1186/s12935-023-02877-y.

## Background

Acute myeloid leukemia (AML) is the most common form of acute leukemia in adults, while it is the second most common form in children [1–3] behind acute lymphoblastic leukemia (ALL) [2, 4].

AML is characterized by genetic mutations and epigenetic dysregulation resulting in a heterogeneous population of malignant cells with blocked differentiation, which causes increased proliferation and self-renewal activity [5, 6]. AML has a poor prognosis and high relapse rate [7]. The primary mechanism of relapse involves leukemia-initiating cells (LICs), a small subpopulation of cells with CD34 + /CD38 − phenotype enrichment that is associated with poor prognosis in AML [7, 8]. LICs have been reported in ALL, but they are less well-defined than LICs in AML [9, 10]. Unlike rapidly dividing AML blasts, dormant LICs are relatively insensitive/resistant to cytotoxic agents [11]. Therefore, developing novel targeted therapies is crucial to eradicate LICs and completely cure AML.

Hematopoietic precursor transformation into LICs can be mediated by the mixed-lineage leukemia 1 (*MLL or MLL1*) fusion oncogene [12, 13]. *MLL1* (also known as lysine methyltransferase 2A [*KMT2A*]) is located on chromosome 11q23, but chromosomal translocation (*MLL1-*rearrangement [*MLL1-r*]) occurs in 5%–10% of acute leukemia cases (AML and ALL) in adults and children [14, 15]. *MLL1* translocations are particularly prevalent in infant leukemias; they are found in up to 80% of infant ALL cases [16]. These translocations create a particularly aggressive subtype of leukemia in children and adults [17]. *MLL1* translocations cause fusion of the N-terminal fragment of *MLL1* with one of > 80 partner proteins, leading to expression of chimeric MLL1 fusion proteins that drive leukemic gene expression and proliferation and block hematopoietic differentiation, resulting in leukemia development [17–21].

The scaffold protein Menin, an essential cofactor, is a highly specific binding partner of MLL and MLL fusion proteins and is required to regulate the target genes of these proteins. Thus, the Menin-MLL1 interaction is critical for initiation and maintenance of acute leukemia [22, 23]. Recent studies have shown the importance of MLL1-Menin interaction in AML with mutated *nucleophosmin 1* (*NPM1c*). *NPM1c* is the most common genetic alteration in adult AML, causing cytoplasmic NPM1 localization in 35.2% of AML patients [24]. The wild-type MLL1-Menin interaction is essential to maintain *NPM1c*-driven leukemia [25].

*MLL1-r* and *NPM1c* leukemias are both associated with upregulated expression of cofactors such as *HOXA, MEIS1* and *PBX3*, which are crucial in leukemia development, cell proliferation, and self-renewal [26]. *MLL1-r* patients respond poorly to currently available treatments [27]. However, small-molecule–mediated inhibition of the protein‒protein interaction between Menin and MLL fusion proteins may help ameliorate *MLL1-r* or *NPM1c* acute leukemia by inhibiting proliferation and inducing differentiation. Recently, highly potent and selective inhibitors of the Menin-MLL interaction such as VTP50469 and MI-3454 have been developed and have shown remarkable preclinical efficacy in *MLL1*-rearranged and *NPM1*-mutated leukemia in vivo models [25, 28–31].

Here we report the development of (1*R*, 2*S*, 4*R*)-4-({[4-(5,6-dimethoxypyridazin-3-yl)phenyl]methyl}amino)-2-{methyl[6-(2,2,2-trifluoroethyl)thieno[2,3-*d*]pyrimidin-4-yl]amino}cyclopentan-1-ol (DS-1594a), a novel, potent, orally available small-molecule inhibitor of the Menin-MLL1 interaction with well-optimized drug properties for the treatment of hematologic malignancy patients, including patients with acute leukemia with *MLL1-r* and *NPM1c* mutations. We aimed to demonstrate the preclinical efficacy of DS-1594a and its salts (DS-1594a·HCl and DS-1594a·succinate) with similar chemical stability in vitro and in vivo using AML/ALL models (cell lines, murine MA9 cells, patient-derived AML/ALL cells, and animal xenografts) although DS-1594a·succinate was selected as the most appropriate formulation for clinical trial (NCT04752163) based on the physicochemical properties. Importantly, we found that DS-1594a·HCl and DS-1594a·succinate mediate eradication of potential LIC fraction by enhancing differentiation and reducing serial colony-forming potential in *MLL1-r* AML cells.

## Methods

### Chemistry

The chemical synthesis of DS-1594a, DS-1594a·HCl, and DS-1594a·succinate is described in the Supplemental Methods. DS-1594a·succinate was selected as the most appropriate formulation for clinical trials in terms of physicochemical properties. There are no critical issues regarding the use of DS-1594a·HCl in nonclinical studies, including issues with chemical stability. The methods used for crystal preparation, crystallographic data collection, and structure determination are described in the Supplemental Methods and Additional file 1: Table S1.

### Biochemical assays

The protocols for human Menin expression and purification, a Menin-MLL1 interaction assay (AlphaLISA), and coimmunoprecipitation experiments are presented in the Supplemental Methods.

### Human MLL-AF9–evoked murine AML-like cells (MA9 cells)

A retroviral expression vector expressing FLAG–MLL-AF9 was prepared as described previously [32]. c-KIT (CD117) + mononuclear bone marrow cells from C57BL/6 J Jms Slc mice (Japan SLC, Inc.) were purified using mouse CD117 MicroBeads (via magnetic-activated cell sorting) and transduced with an MLL-AF9 retrovirus on a RetroNectin (TaKaRa)-coated nontreated 6-well plate.

The methods used for May-Grünwald-Giemsa (MGG) staining, a colony-forming unit assay using MethoCult medium, and a cellular growth assay in murine MA9 cells are described in the Supplemental Methods.

### Cell lines and patient-derived primary AML cells

The human leukemic cell lines MV-4–11 (ATCC) and K-562 (ATCC) were cultured in Iscove’s modified Dulbecco’s medium (IMDM) with 10% (v/v) fetal bovine serum (FBS); HL-60 (ATCC) was cultured in IMDM with 20% (v/v) FBS; KOPN-8 (DSMZ), KG-1 (ATCC) and MOLM-13 (DSMZ) were cultured in in RPMI-1640 (Invitrogen) with 10% (v/v) FBS; OCI-AML3 (DSMZ) was cultured in alpha minimal essential medium (Gibco) with 20% (v/v) FBS; and OCI-AML5 (DSMZ) was cultured in alpha minimal essential medium with 20% (v/v) FBS and 10 ng/mL granulocyte–macrophage colony-stimulating factor (PeproTech). Adult patient-derived AML#8531 (*MLL1-r* AML) and AML676 (*MLL1-r* AML) cells were kindly provided by CRB Hospices Civils de Lyon and purchased from ProteoGenex. Inc., respectively. Pediatric patient-derived NCCHD010 (*MLL1-r* AML) and NCCHD007 (*MLL1-r* ALL) cells were kindly provided by the National Center for Child Health and Development (NCCHD). The methods used for the cell viability assay and staining in human leukemic cell lines are described in the Figure Legends and Supplemental Methods.

The protocols for flow cytometry (FCM) analysis for murine MA9 cells and patient-derived AML cells, RNA purification and real-time quantitative polymerase chain reaction (RT‒qPCR), cDNA library preparation for RNA sequencing (RNA-seq) and whole-transcriptome analysis, chromatin immunoprecipitation (ChIP), and library preparation for ChIP sequencing (ChIP-seq) and analysis are also described in the Figure Legends and Supplemental Methods.

The nucleotide sequence data have been deposited in the DNA Data Bank of Japan (accession number JGAS000511). The Protein Data Bank registration number for the X-ray cocrystal data is PDB 8IG0.

### Animals

Four- to six-week-old NOD scid gamma (NSG; NOD. Cg-Prkdcscid Il2rgtm1Wjl/SzJ) mice were purchased from Japan Charles River Co., Ltd. Four- to six-week-old NOD/SCID (nonobese-diabetic Prkdcscid) mice were purchased from Beijing Anikeeper Biotech Co., Ltd. All in vivo studies were approved by the Institutional Animal Care and Use Committee (IACUC) of Daiichi Sankyo Co., Ltd. The animal models, survival experiments, and hematoxylin and eosin staining methods are described in the Figure Legends and Supplemental Methods.

All animal experiments were approved by the IACUC of Daiichi Sankyo Co., Ltd. The experimental data management and reporting procedures strictly adhered to the applicable Crown Bioscience, Inc., Guidelines and Standard Operating Procedures.

### Statistics

SAS System Release 9.2 (SAS Institute, Inc.) was used for statistical analyses. Significant differences in survival curves were analyzed using the Kaplan‒Meier log-rank test, with an increased survival time corroborating a test compound’s life-prolonging effect. For gene set enrichment analysis (GSEA), the t test was used to score and rank genes. Each gene set’s *FDR q* value was calculated by gene set permutation.

### Data sharing statement

For original data, please contact numata.masashi.ak@daiichisankyo.co.jp.

## Results

### Development of a highly potent Menin-MLL1 inhibitor

**Fig. 1:**
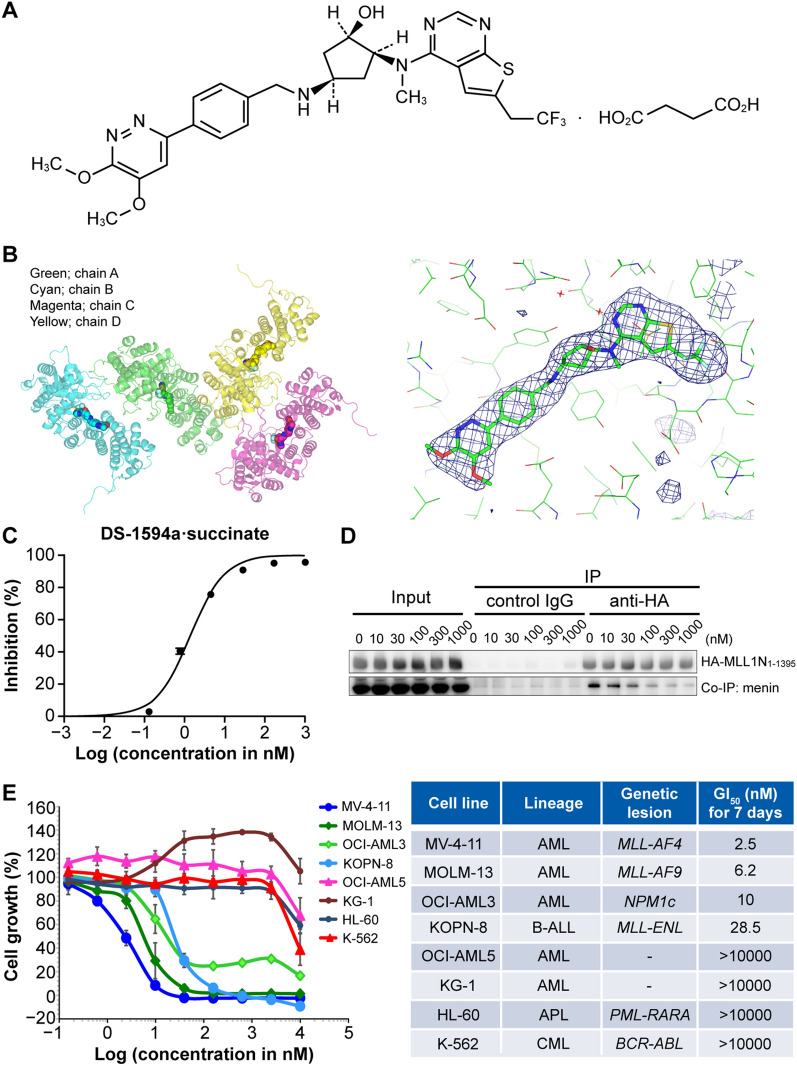
Structure and in vitro activity of the Menin-MLL1 inhibitor DS-1594a·succinate. **A** Chemical structure of DS-1594a·succinate (succinic acid salt of DS-1594a). **B** Overall structure of the DS-1594a·succinate/human Menin complex showing 4 chains of Menin included in 1 asymmetric unit (left) and Fo-Fc electron density map of DS-1594a·succinate bound to chain A of Menin contoured at 3 sigma (right). **C** Inhibition of the Menin-MLL1 interaction by DS-1594a·succinate in a cell-free assay (AlphaLISA assay). The inhibition (%) of the Menin-MLL1 interaction by DS-1594a·succinate is shown as the mean ± SD at each concentration of DS-1594a·succinate (1000, 170, 28, 4.6, 0.77, 0.13, 0.021, and 0.0036 nM; n = 4). **D** Inhibition of the Menin-MLL1 interaction by DS-1594a·succinate in a cellular assay. Co-immunoprecipitation (Co-IP) was performed with an anti-HA antibody or control IgG in human embryonic kidney 293 T (HEK293T) cells transfected with HA-MLL1N_1-1395_ upon treatment with DMSO (0) or DS-1594a·succinate (10, 30, 100, 300, 1000 nM). The input represents lysate samples before Co-IP treatment. **E** In vitro growth-inhibitory effects of DS-1594a·HCl (the HCl salt of DS-1594a) against leukemia cell lines (left). Titration curves from the cell viability assay (CellTiter-Glo) were created after 7 days of treatment of human leukemic cell lines with DS-1594a·HCl. The horizontal axis is the common logarithm of the concentration of DS-1594a·HCl, and the mean (± SD) survival rates at each concentration are shown (n = 3). The GI_50_ concentrations of DS-1594a·HCl for each cell line are shown (right). B-ALL, B-cell acute lymphoblastic leukemia; CML, chronic myeloid leukemia; APL, acute promyelocytic leukemia; Co-IP, coimmunoprecipitation

Through high throughput screening (HTS) and derivatization from the lead scaffolds, we developed DS-1594a·succinate (the succinic acid salt of DS-1594a; (1*R*, 2*S*, 4*R*)-4-({[4-(5,6-dimethoxypyridazin-3-yl)phenyl]methyl}amino)-2-{methyl[6-(2,2,2-trifluoroethyl)thieno[2,3-*d*]pyrimidin-4-yl]amino}cyclopentan-1-ol; Fig. 1A), which contained unique chemical structures such as (1*R*, 2*S*, 4*R*)-cyclopentan-1-ol and 5,6-dimethoxypyridazine moieties compared with those of other Menin-MLL1 inhibitors [33, 34]. These structures enabled DS-1594a·succinate to display the most favorable drug-like properties among our derivatives and potent inhibitory activity against the Menin-MLL1 interaction. For crystallographic study of the interaction of DS-1594a·succinate with Menin, human Menin with deletion of residues 460–519 was expressed in *Escherichia coli* and purified as described previously with some modifications [35]. Thereafter, the crystal structure of Menin bound to DS-1594a·succinate was solved to reveal the molecular basis of DS-1594a·succinate-mediated inhibition (Fig. 1B). The structure contains 4 molecules in the asymmetric unit. However, we focused on the chain A interaction mode, as no significant interaction mode changes were observed among chains A-D. The structure demonstrated a number of hydrophobic interactions between DS-1594a·succinate and surrounding amino acids that likely contributed to DS-1594a·succinate’s strong inhibitory activity. It also demonstrated that DS-1594a·succinate forms a hydrogen bond with Tyr276 and engages in π-π or CH-π interactions with Phe238, Met278, Tyr319, Met322, Tyr323, and Trp341 (Additional file 1: Figure S1A), which likely contributes to its target specificity. Notably, the 5,6-dimethoxypyridazine moiety seems to contribute to both inhibitory activity and target specificity through the formation of typical CH-π interactions with the Trp341 side chain (Fig. 1C) and through extremely good shape complementarity with the surrounding pocket (Additional file 1: Figure S1B).

DS-1594a·succinate demonstrated inhibitory activity against the Menin-MLL1 interaction in the subnanomolar range (with a half-maximal inhibitory concentration of 1.4 nM) in a cell-free AlphaLISA assay using a full-length recombinant Menin protein and MLL1 peptide (Fig. 1D and Additional file 2). DS-1594a·succinate was characterized in detail in human embryonic kidney cells (HEK293T) expressing an HA-human MLL1​N​ truncated protein (HA-MLL1N_1-1395_) to assess its ability to block the Menin-MLL1 interaction, as indicated by coimmunoprecipitation of Menin and HA-MLL1N_1-1395_. Our results demonstrated that DS-1594a·succinate could reach the target protein and inhibit endogenous Menin protein and the exogeneous HA-MLL1N_1-1395_ interaction at more than 30 nM in a dose-dependent manner (Fig. 1E).

Thereafter, we tested DS-1594a·HCl (HCl salt of DS-1594a) activity in leukemic cell lines with and without *MLL1-r* and *NPM1c*. DS-1594a·HCl markedly inhibited the cellular growth of leukemic cells harboring various MLL fusion proteins (MV-4–11, MOLM-13, KOPN-8) and *NPM1c* (OCI-AML3), with 50% growth inhibition (GI_50_) values between 2.5 and 28.5 nM. In contrast, DS-1594a·HCl did not inhibit leukemic cell growth (exhibiting > 350-fold selectivity) without *MLL1-r* or *NPM1c* (OCI-AML5, KG-1, HL-60 and K-562; Fig. 1F).

### The Menin-MLL1 inhibitor induced differentiation and loss of clonogenic potential of human MLL-AF9–evoked murine AML-like cells

**Fig. 2:**
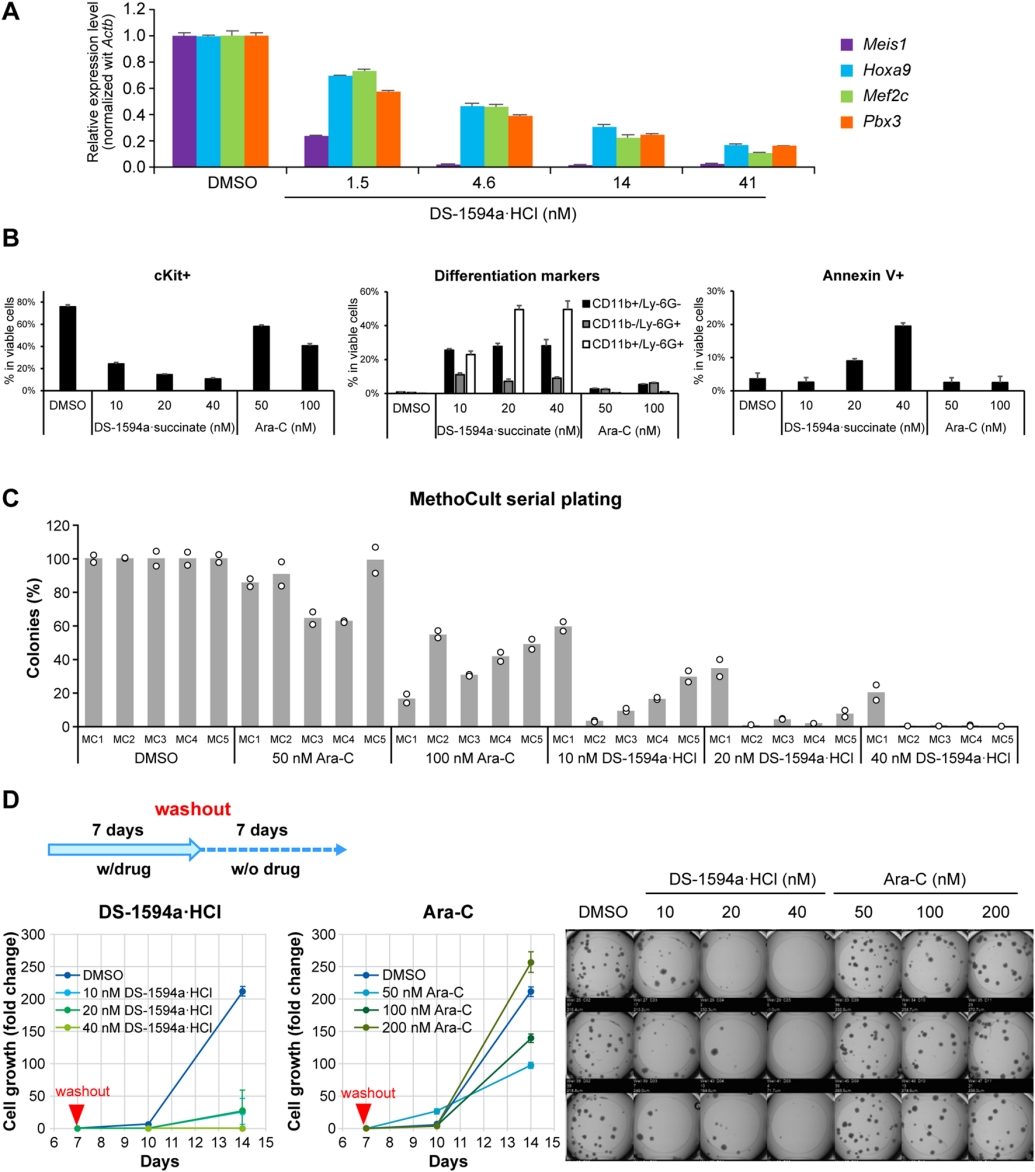
The Menin-MLL1 inhibitor induced differentiation and loss of LICs of human MLL-AF9–evoked murine AML-like cells **A** RT‒qPCR was performed in *MLL-AF9*–evoked murine AML-like cells after treatment with DS-1594a·HCl (1.5, 4.6, 14, and 41 nM) for 4 days. The expression levels of *Meis1, Hoxa9, Mef2c*, and *Pbx3* were normalized to that of *Actb* and compared to those in the DMSO-treated control (mean ± SD; n = 3). **B** FCM analysis with the indicated antibodies (cKit, CD11b, Ly-6G, and Annexin V) for *MLL-AF9–*evoked murine AML-like cells after 7 days of treatment with DMSO, DS-1594a·succinate (10, 20, and 40 nM), or Ara-C (50 and 100 nM). The bars represent the mean ± SD; n = 3. **C** Serial replating in the MethoCult M3434 assay. The colonies were counted 4–5 days after seeding with DMSO, DS-1594a·HCl (10, 20, and 40 nM), or Ara-C (50 and 100 nM). The first colony (MC1) was counted, collected, and replated (MC2–MC5) in fresh MethoCult M3434 with DMSO or test compounds (n = 2, represented by each circle). **D** The cellular growth and colony formation activity (MethoCult M3434 assay) of *MLL-AF9–*evoked murine AML-like cells were monitored after washout of DS-1594a·HCl or Ara-C pretreatment for 7 days. Ara-C, cytarabine

We evaluated the effects of DS-1594a·HCl and DS-1594a·succinate on differentiation and population of leukemia-initiating cell (LIC) fractions in *MLL1-r* AML-like cells, using RT‒qPCR, MGG staining, FCM, serial colony formation and growth assays. MLLfusion target gene expression in human *MLL-AF9*–evoked murine AML-like (MA9) cells was assessed after 4 days of treatment with DS-1594a·HCl. RT‒qPCR confirmed that DS-1594a·HCl exhibited concentration-dependent inhibition of *Meis1*, *Hoxa9*, *Mef2c*, and *Pbx3* expression (Fig. 2A).

This downregulation of MLL-fusion target gene expression occurred with an increase in the number of differentiated cells on microscopic examination of MGG–stained murine MA9 cells. The number of cells differentiated into granulocytic lineages with lobulated cell nuclei and loss of cytoplasmic color increased in a DS-1594a·HCl concentration-dependent manner at final concentrations of 10, 20, and 40 nM for 7 days, in contrast to the findings among cells treated with dimethyl sulfoxide (DMSO) or standard chemotherapy drug cytarabine (Ara-C; Additional file 1: Figure S2A). The proportion and extent of differentiation induction were assessed by counting of the differentiated and undifferentiated cells. Compared with the percentage after treatment with DMSO (control; 87%), the percentage of blasts, the most undifferentiated cell type, decreased in a concentration-dependent manner to 51%, 20%, and 5.7% after treatment with 10, 20, and 40 nM DS-1594a·HCl, respectively. Furthermore, the percentage of neutrophils, the most differentiated cell type, increased in a concentration-dependent manner to 1.4%, 6%, and 33% after treatment with 10, 20, and 40 nM DS-1594a·HCl, respectively, while the percentage remained 0% after treatment with DMSO (control, 0%; Additional file 1: Figure S2B).

FCM analysis of murine MA9 cells treated with DMSO, DS-1594a·succinate, or Ara-C for 7 days was performed with antibodies against the LIC-associated marker c-KIT [12, 36], the granulocytic and myeloid differentiation markers CD11b and Ly-6G, and the apoptosis marker Annexin V (Fig. 2B, Additional file 1: Figure S2C, S2D and S2E). Our results showed that the CD11b + Ly-6G + and Annexin V + fractions increased by 50% and 19%, respectively, with 40 nM DS-1594a·succinate, while the c-KIT + fraction decreased by 11%. No significant increases or decreases in the CD11b + Ly-6G + , c-KIT + , and Annexin V + fractions were observed with 100 nM Ara-C, the standard-of-care drug for AML treatment, compared with the DMSO control.

The colony-forming and self-replicating ability of murine MA9 cells was tested using a MethoCult assay. Unlike for DMSO-treated cells (100%), no colonies were detected in the fifth serial replating of 40 nM DS-1594a·HCl-treated cells, while ⁓50% of colonies were detected in 100 nM Ara-C-treated cells (Fig. 2C). To verify the durable efficacy after DS-1594a·HCl treatment termination, MethoCult assays were performed after compound washout in murine MA9 cells treated with DMSO, DS-1594a·HCl, or Ara-C for 7 days. On day 7 after washout (day 14), the proliferative and colony-forming ability of the 40 nM DS-1594a·HCl–treated cells was eliminated, whereas that of the 200 nM Ara-C–treated cells was only slightly suppressed and not suppressed compared with that of the DMSO-treated cells on days 10 and 14, respectively (Fig. 2D). Overall, our results indicate that the Menin-MLL1 inhibitor might deplete cKit + fractions reflecting LICs via strong differentiation-inducing activity with suppression of Menin-MLL1 regulatory gene expression even at very low concentrations.

### The Menin-MLL1 inhibitor induced differentiation and loss of CD34 + /CD38 − cells in patient-derived MLL1-r + AML cells (AML#8531, AML676, and NCCHD010) in vitro

**Fig. 3:**
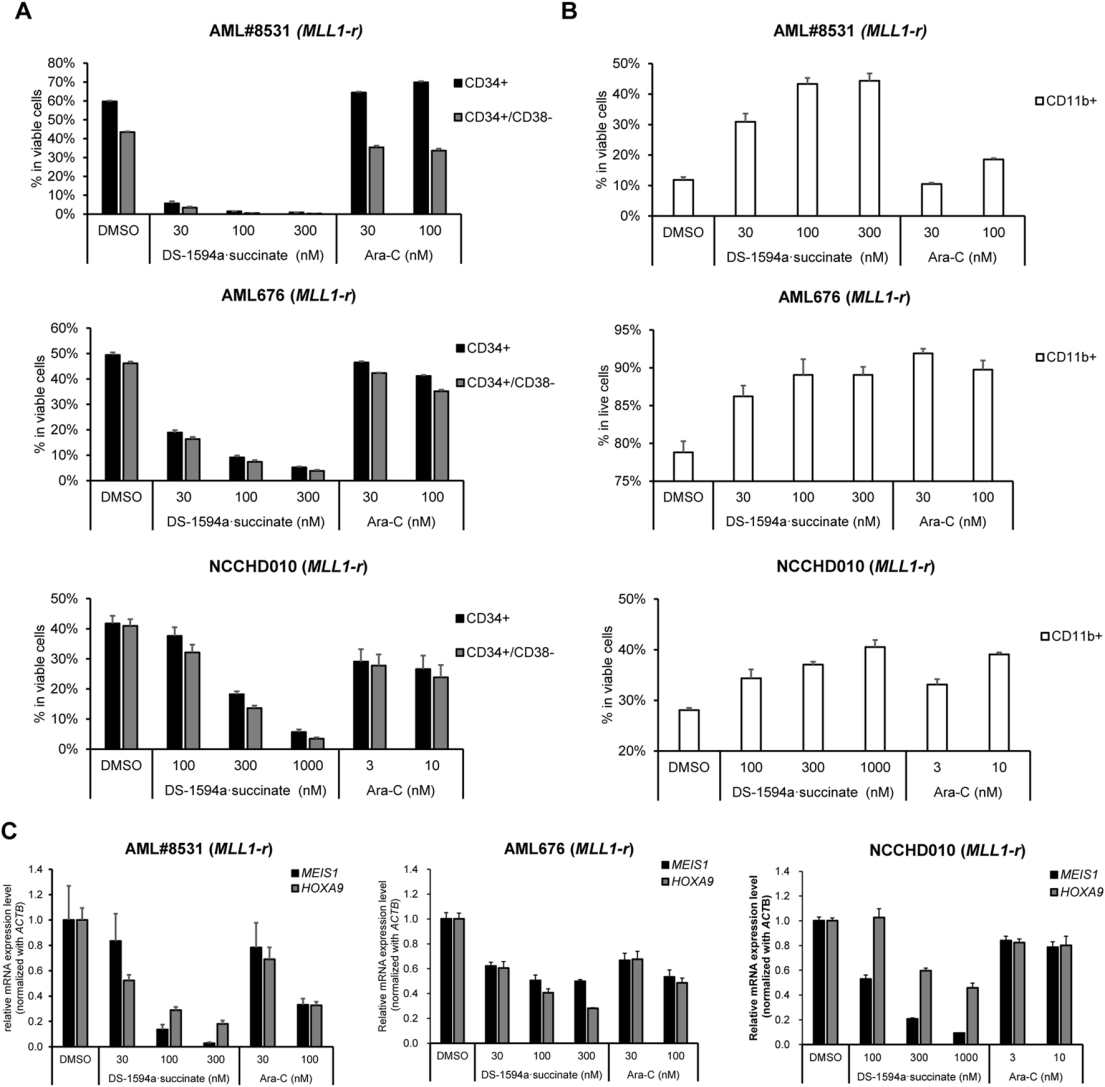
The Menin-MLL1 inhibitor induced differentiation and loss of CD34 + /CD38 − cells in patient-derived MLL1-r + AML cells (AML#8531, AML676, and NCCHD010) in vitro. Patient-derived primary AML cells (*MLL1-r* AML#8531, NCCHD010, AML676) were treated for 7 days with DMSO, DS-1594a·succinate, or Ara-C at the indicated concentrations.** A** FCM analysis with anti-human CD34/CD38 antibodies. The bars represent the mean ± SD; n = 3. **B** FCM analysis with anti-human CD11b. The bars represent the mean ± SD; n = 3. **C** RT‒qPCR to detect the expression levels of *MEIS1* and *HOXA9* (mean ± SD; n = 3) normalized to that of *ACTB*

We evaluated the effect of DS-1594a·succinate on induction of differentiation and loss of CD34 + /CD38 − cells, which are a fraction of potential LICs, in patient-derived *MLL1*-r + AML cells (AML#8531, AML676, and NCCHD010) in vitro. Compared with DMSO, DS-1594a·succinate induced strong CD34 downregulation in a dose-dependent manner, leading to decreased CD34 + /CD38 − cell frequencies (Fig. 3A and Additional file 1: Figure S3A). Consistent with the loss of the CD34 + /CD38 − fraction, an increased proportion of cells expressing the monocytic differentiation marker CD11b was observed after DS-1594a·succinate in AML#8531, AML676 and NCCHD010 cells (Fig. 3B and Additional file 1: Figure S3B). The DS-1594a·succinate-mediated downregulation of CD34 and upregulation of CD11b were simultaneously confirmed by RT‒qPCR in AML#8531 and AML676 cells (Additional file 1: Figure S3C).

In parallel, DS-1594a·succinate greatly reduced expression of MLL-fusion target genes, *MEIS1* and *HOXA9*, dose dependently in patient-derived *MLL1-r* + AML cells (Fig. 3C), as in *NPM1c* AML patient-derived cells (AML#7789, AML#7915, AML#7919; Additional file 1: Figure S4). These results suggest that enhancement of differentiation and potential loss of LIC fraction via reductions in *MEIS1* and *HOXA9* expression are major mechanisms of action for DS-1594a antitumor activity.

### The Menin-MLL1 inhibitor induced gene expression changes through Menin-MLL1 chromatin-associated complexes

**Fig. 4:**
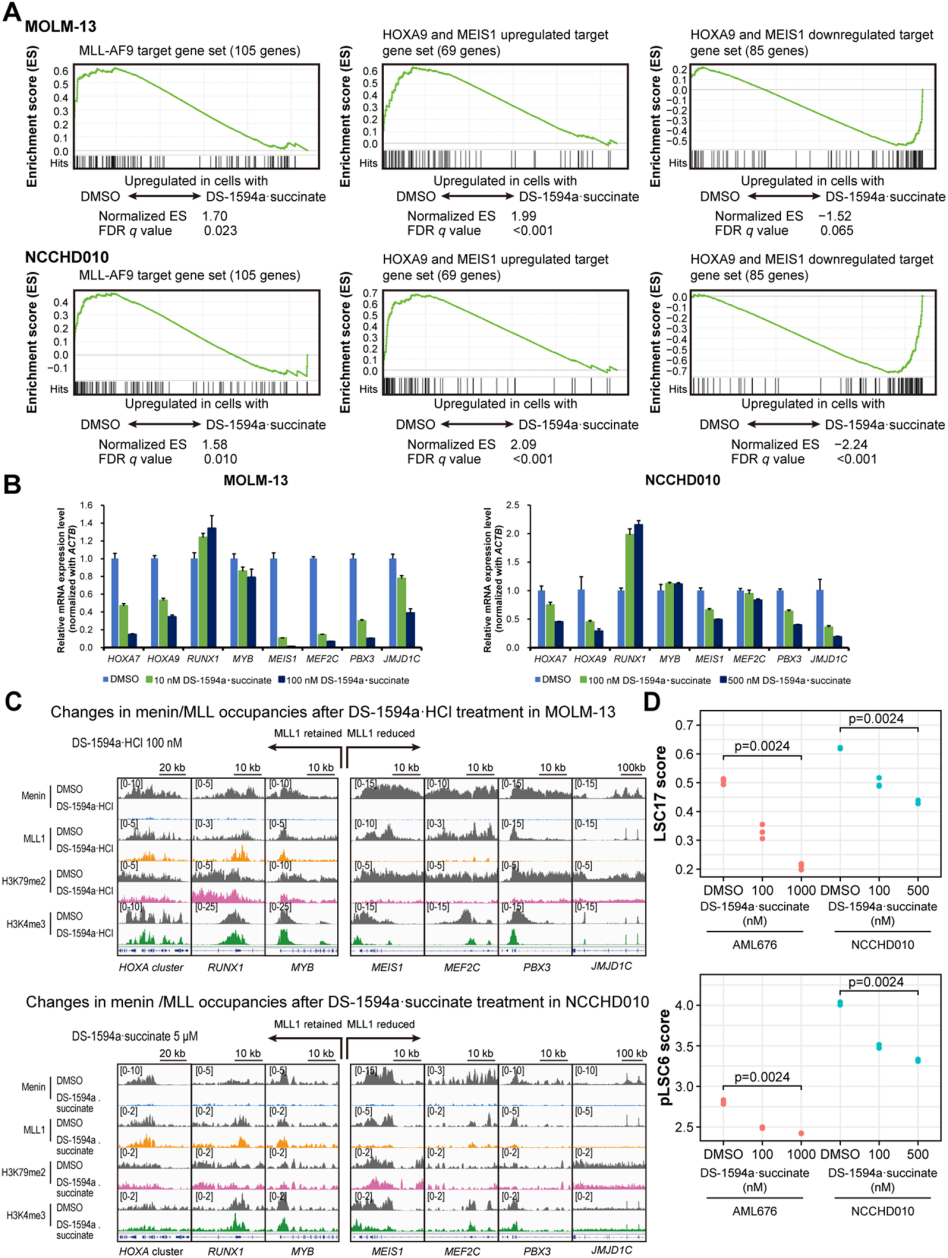
The Menin-MLL1 inhibitor induced gene expression changes through Menin-MLL1 chromatin-associated complexes. **A** RNA-seq was performed in MOLM-13 and NCCHD010 cells treated with DS-1594a·succinate for 3 and 7 days, respectively. GSEA of MOLM-13 cells treated with 100 nM DS-1594a·succinate for 3 days and NCCHD010 cells treated with 500 nM DS-1594a·succinate for 7 days. **B** RT‒qPCR was performed in MOLM-13 and NCCHD010 cells treated with DS-1594a·succinate for 3 and 7 days, respectively, at the indicated concentrations. The graphs represent the expression levels of the indicated genes compared to those in the DMSO-treated control (mean ± SD, n = 3). **C** Changes in Menin/MLL1 occupancies in MOLM-13 and NCCHD010 cells treated with 100 nM or 5 μM DS-1594a·HCl or DS-1594a·succinate, respectively, for 3 days. Normalized coverage tracks of Menin, MLL1, H3K79me2, and H3K4me3 ChIP-seq signals (reads per million) at selected target genes in MOLM-13 or NCCHD010 cells are shown. The peaks around the TSS are shown. **D** Analysis of the leukemic stem cell score. LSC17 scores and pLSC6 scores in AML676 and NCCHD010 cells were calculated for each sample treated with DMSO or DS-1594a·succinate at the indicated concentrations (n = 3). The numbers on the plots are *P* values of the Jonckheere-Terpstra trend test with Bonferroni correction. For the GSEA, the t test results were used as the metrics for scoring and ranking genes. The FDR q value for each gene set was calculated with the permutation of the gene sets. LSC17, 17-gene adult LSC; pLSC6, 6-gene pediatric LSC; TSS, transcription start site

To investigate the effects of Menin-MLL inhibitors on global gene expression in *MLL1*-rearranged leukemias, we performed RNA-seq with RNA from a human *MLL1*-r AML cell line (MOLM-13) and patient-derived primary AML with *MLL1*-r (NCCHD010) cells treated with DS-1594a·succinate for 3 and 7 days, respectively. Significant gene expression changes were found for 560 vs 186 genes and 737 vs 271 genes were ≥ twofold upregulated vs downregulated in MOLM-13 (100 nM DS-1594a·succinate) and NCCHD010 (500 nM DS-1594a·succinate) cells (p ≤ 0.05). GSEA was also used to determine changes in MLL-fusion target–related signature expression in DS-1594a·succinate–treated MOLM-13 and NCCHD010 cells. We used the gene list from prior publications; of 139 listed *MLL-AF9* target gene members [37], 105 genes that could be mapped in humans were used, and 69 and 85 target genes that were upregulated and downregulated, respectively, in hematopoietic precursor cells conditionally expressing *HOXA9* and *MEIS1* were used [38, 39]. GSEA demonstrated strong downregulation of *MLL-AF9* downstream targets and *HOXA9-* and *MEIS1*-upregulated genes with DS-1594a·succinate in MOLM-13 and NCCHD010 cells. GSEA also showed upregulation of *HOXA9-* and *MEIS1-*downregulated genes in MOLM-13 and NCCHD010 cells (Fig. 4A). DS-1594a·succinate concentration-dependently reduced the expression of various MLL-fusion target genes, including *MEIS1*, *MEF2C*, *PBX3, JMJD1C*, *HOXA7*, and *HOXA9,* in RNA-seq studies (Additional file 1: Figure S5A), and these gene expression changes were validated by RT‒qPCR (Fig. 4B).

As DS-1594a·HCl and DS-1594a·succinate treatment disrupted Menin-associated protein complexes and MLL fusion-driven gene expression, we assessed the chromatin occupancy of Menin and MLL1 proteins and histone modifications important in *MLL1-r* leukemia, including H3K79me2 and H3K4me3. ChIP-seq was performed on the genomic binding sites and the dynamics of Menin and MLL1 interactions with chromatin after DS-1594a·HCl or DS-1594a·succinate treatment (3 days) in MOLM-13 (100 nM DS-1594a·HCl) and NCCHD010 (500 nM or 5 μM DS-1594a·succinate) cells. Menin binding analysis confirmed that DS-1594a·HCl and DS-1594a·succinate treatment led to local loss of Menin binding to chromatin. The sites and magnitude of Menin loss were similar between MOLM-13 and NCCHD010 cells. We observed reduced levels of MLL1, H3K79me2, and H3K4me3 at genes including *MEF2C, MEIS1*, *PBX3*, and *JMJD1C*, but not at the *HOXA* genes *RUNX1* and *MYB* (Fig. 4C; Additional file 1: Figure S5B). Together, the data suggest that only a subset of MLL target genes are sensitive to inhibition of Menin, possibly by either genetic deletion or pharmacologic perturbation.

Finally, we evaluated stemness/LSC scores to assess whether DS-1594a·succinate impairs LSC-enriched gene expression in patient-derived primary *MLL1-r* AML cells (AML676, NCCHD010). Adult and pediatric stemness scores, assessed by 17-gene adult LSC (LSC17) and 6-gene pediatric LSC (pLSC6) scores, respectively, were significantly decreased in DS-1594a·succinate–treated cells, indicating the biological impact on potential fractions of LSCs or LICs (Fig. 4D).

### The Menin-MLL1 inhibitor showed in vivo antitumor efficacy in a MOLM-13 xenograft model

**Fig. 5:**
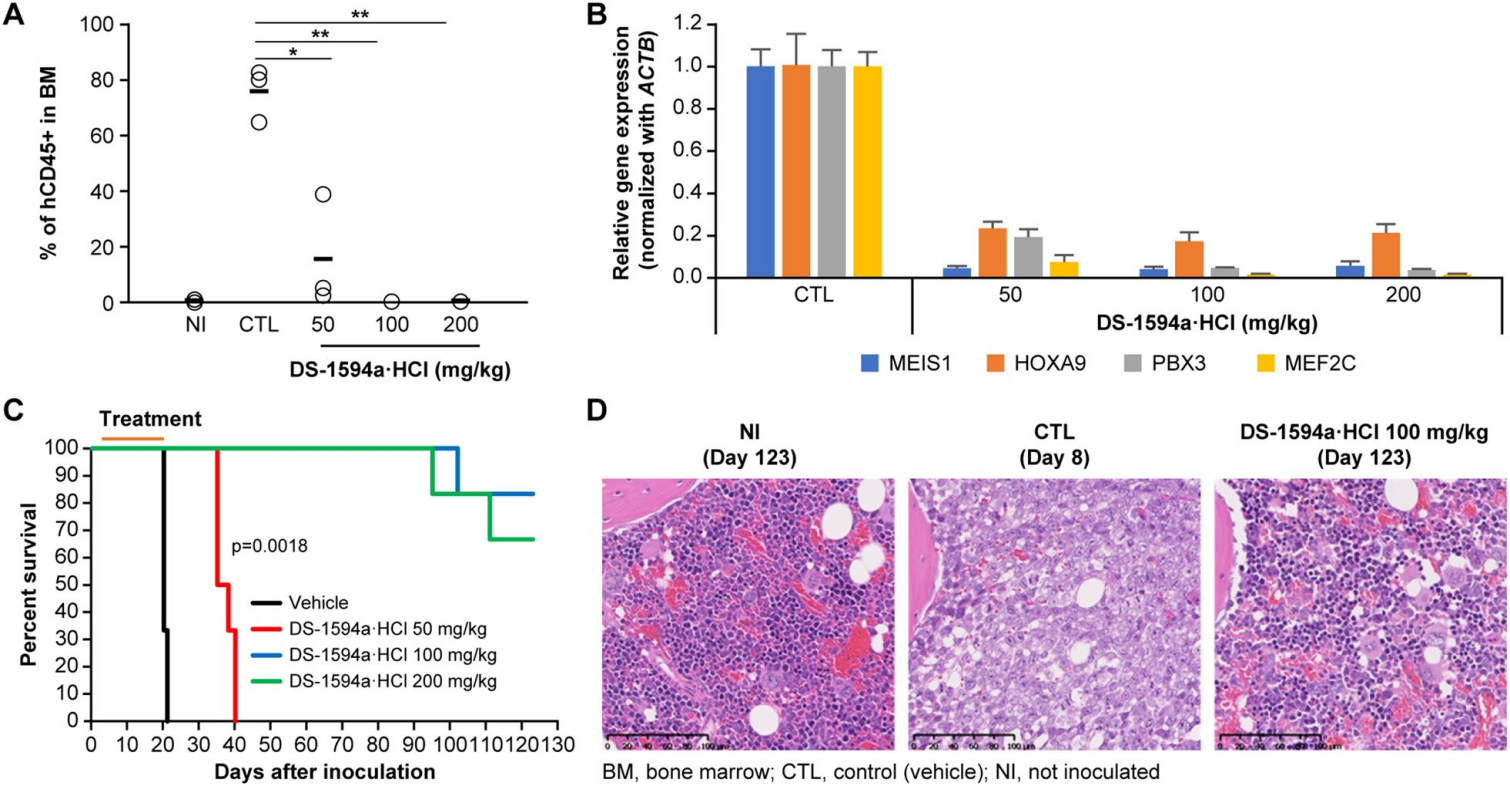
The Menin-MLL1 inhibitor showed in vivo antitumor efficacy in a MOLM-13 xenograft model.** A** FCM analysis to assess tumor burden (hCD45 +) in BM 1 day after 17 days of treatment (days 3–19). BM cells from NI NSG mice or NSG mice intravenously inoculated with MOLM-13 cells 1 day after 17-day treatment (day 20) with vehicle (CTL, QD × 17) or DS-1594a·HCl (50, 100, 200 mg/kg, QD × 17). The horizontal bars represent the mean tumor burden (n = 3 mice per group). *p = 0.0005, **p < 0.0001 (Dunnett’s test). **B** RT‒qPCR was performed for BM cells from MOLM-13 xenografts 6 h after dosing with vehicle (CTL, QD × 7) or DS-1594a·HCl (50, 100, 200 mg/kg, QD × 7). The expression levels of *MEIS1*, *HOXA9*, *PBX3* and *MEF2C* were normalized to that of *ACTB* (mean ± SD, n = 3). **C**)Kaplan–Meier survival curves of vehicle- or DS-1594a·HCl–treated MOLM-13 xenograft mice (days 3–21, QD × 19; n = 6 mice per group). **D** Representative images from H&E staining of BM from NI mice (day 123), CTL mice (day 8), and 100 mg/kg DS-1594a·HCl-treated mice (day 123). The significance of the survival curves was analyzed by the Kaplan‒Meier log-rank test, with survival time as the indicator of the life-prolonging effect of the test compound.QD, once daily

DS-1594a·HCl’s in vivo antitumor efficacy was investigated using an intravenously inoculated *MLL1-r* AML leukemia xenograft model. MOLM-13 cells were transplanted into NSG mice to induce systemic leukemia, and disease progression was monitored. FCM analysis of bone marrow cells from the hindlimb bones showed that the human CD45 (hCD45) positivity rate in myeloid cells was ~ 76% in the vehicle group vs. ~ 16% in the 50 mg/kg DS-1594a·HCl group. Furthermore, the percentages of hCD45 + cells in the 100 and 200 mg/kg DS-1594a·HCl groups decreased to ≤ 1% (Fig. 5A).

RT‒qPCR analysis of RNA from hindlimb bone marrow cells revealed that the DS-1594a·HCl–treated group showed reductions in the expression of Menin-MLL1-regulated genes such as *MEIS1, HOXA9, PBX3*, and *MEF2C* by up to 96%, 83%, 96%, and 99%, respectively (Fig. 5B). These results indicate that DS-1594a·HCl suppresses the expression of Menin-MLL1–regulated genes in MOLM-13 cells in murine bone marrow, which is correlated with repression of MOLM-13 cell growth.

The persistence of DS-1594a·HCl–mediated AML cell growth inhibition in MOLM-13 xenograft mice was evaluated using survival tests. In the vehicle group, all mice died within 21 days of transplantation. In contrast, the DS-1594a·HCl 50, 100, and 200 mg/kg-treated groups showed a significantly pronounced survival benefit after treatment termination (increase in life span [ILS]: 82.5%, > 515%, > 515%, p = 0.0018; Fig. 5C). DS-1594a·HCl-treated mice (100 and 200 mg/kg) that were alive 123 days after transplantation remained in good condition without weight loss at all times after treatment termination. In these mice, the hCD45 positivity rate in the bone marrow for the 100 mg/kg DS-1594a·HCl dosage group was ≤ 1% and similar to that in the non-inoculated (NI) group (data not shown), suggesting that MOLM-13 cells may have been completely eradicated from the bone marrow.

In the control (vehicle-treated) MOLM-13 xenograft group, the femoral bone marrow cavities of all mice (n = 3) contained predominantly neoplastic cells, with minimal residual normal bone marrow. Tumor cell proliferation was also detected between the cortical bone and periosteum on the distal end. However, no neoplastic cells were found in the femoral marrow cavities of any mice that survived (n = 5) at day 123 in the DS-1594a·HCl 100 mg/kg group, as in the femurs in the NI group, indicating similar normal marrow status (Fig. 5D).

### The Menin-MLL1 inhibitor showed in vivo antitumor efficacy in AML-ALL PDX models

**Fig. 6:**
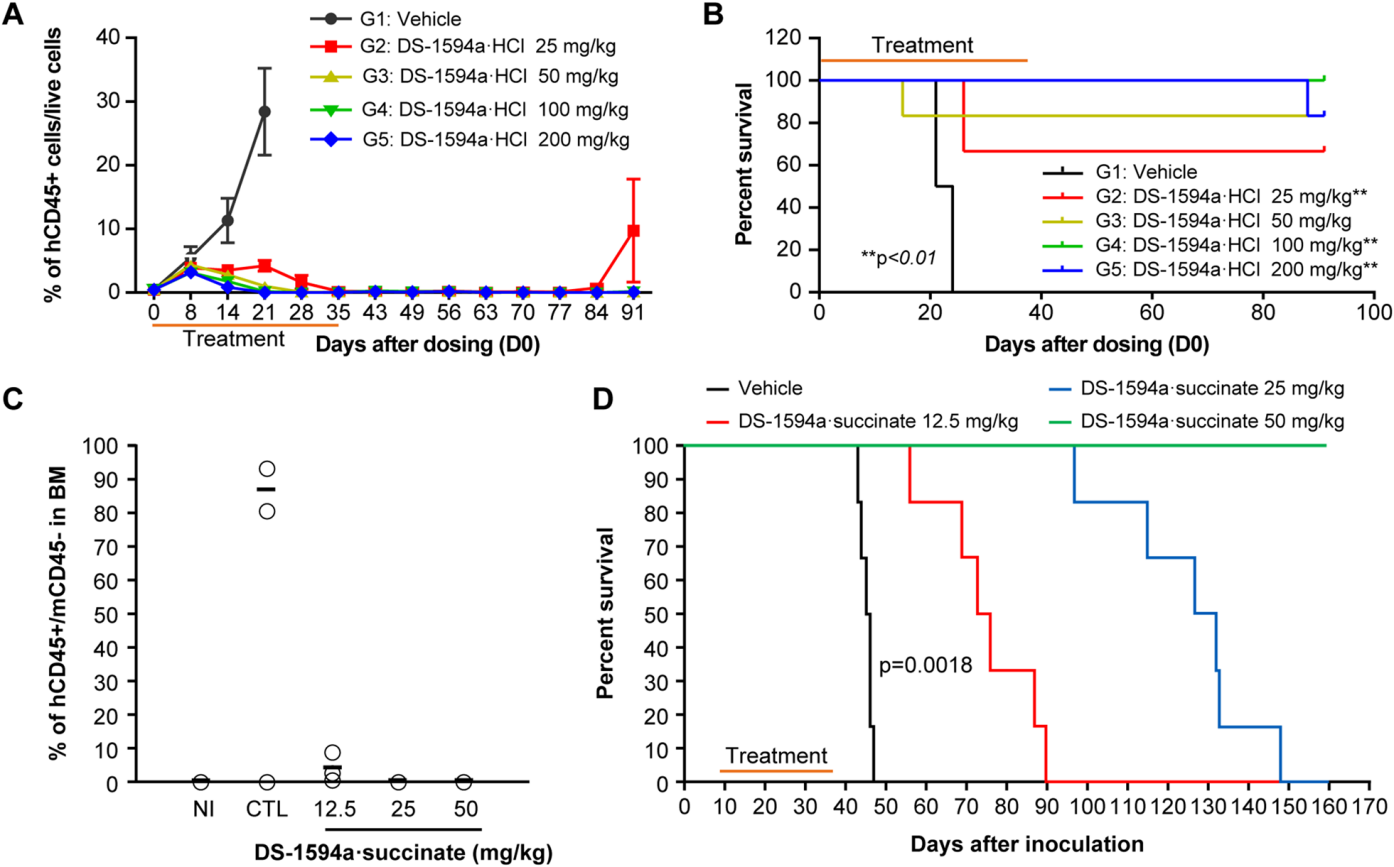
The Menin-MLL1 inhibitor showed in vivo antitumor efficacy in AML-PDX (**A**-**B**) and ALL-PDX (**C**-**D**) models. **A** FCM analysis to demonstrate the kinetics of tumor burden (percentage of hCD45 + cells) in PB from AM7577 mice 31 days after inoculation (vehicle [days 0–23, QD × 24] or DS-1594a·HCl [25, 50, 100, and 200 mg/kg; days 0–34, QD × 35], n = 6 mice per group). **B** Kaplan‒Meier survival curves of AM7577 mice (vehicle [days 0–23, QD × 24] or DS-1594a·HCl [25, 50, 100, and 200 mg/kg; days 0–34, QD × 35], n = 6 mice per group). **C** FCM analysis to assess tumor burden (percentage of hCD45 + cells) 1 day after 28 days of treatment (day 38) in the BM of NCCHD007 mice (vehicle or 12.5, 25, and 50 mg/kg DS-1594a·succinate, n = 3 mice per group). **D** Kaplan‒Meier survival curves of NCCHD007 mice (vehicle or 12.5, 25, and 50 mg/kg DS-1594a·succinate; days 10–37, BID × 28, n = 6 mice per group). The significance of the survival curves was analyzed by the Kaplan‒Meier log-rank test, with survival time as the indicator of the life-prolonging effect of the test compound. BID, twice daily; mCD, mouse CD; NI, non-inoculated; PB, peripheral blood; QD, once daily

The results obtained with MOLM-13 xenografts prompted further investigation into the efficacy of the Menin-MLL1 inhibitor on patient-derived xenograft (PDX) models. In vivo antitumor efficacy of DS-1594a·HCl in an *NPM1c* AMLPDX model (AM7577) was evaluated by treating with vehicle or DS-1594a·HCl (25, 50, 100, or 200 mg/kg) for 24 and 35 days, respectively. DS-1594a·HCl dosing groups showed complete elimination of hCD45 + AML blast cells in peripheral blood, which was sustained for 40 days after the dosing termination on day 35 (Fig. 6A). Similarly, DS-1594a·HCl induced significant reductions in the proportions of hCD45 + cells in the peripheral blood, spleen, and bone marrow in the DS-1594a·HCl group vs. the vehicle group (p < 0.05; Additional file 1: Figure S6) at the time of mouse termination. Survival was significantly prolonged in the DS-1594a·HCl 25 mg/kg group vs. the vehicle group (p < 0.01, ILS > 296%; Fig. 6B). No body weight loss was observed in the vehicle group or in the DS-1594a·HCl groups for any doses (data not shown).

DS-1594a·succinate’s in vivo antitumor efficacy in an *MLL1-r* ALL-PDX model (NCCHD007) was evaluated after the treatment for 28 days. On day 38, the hCD45 positivity rate in hindlimb bone marrow cells in NCCHD007 mice was ~ 87% in the vehicle-treated group vs. 4%, 0.19%, and 0.21% in the DS-1594a·succinate 12.5, 25, and 50 mg/kg groups, respectively; the latter two rates were close to the background level in the NI group (~ 0.11%; Fig. 6C). The vehicle-treated group showed ~ 18% hCD45 positivity in peripheral blood cells, while the DS-1594a·succinate groups (12.5, 25, and 50 mg/kg) showed ~ 0.00%–0.02% hCD45 positivity, which was comparable to the ~ 0.02% background level in the NI group (data not shown). Furthermore, RT‒qPCR analysis of bone marrow cells from day 38 showed that compared to the vehicle, DS-1594a·succinate markedly reduced the mRNA levels of the downstream MLL1-fusion target genes *MEIS1* and *MEF2C* (Additional file 1: Figure S7).

Moreover, vehicle-treated NCCHD007 mice were dead within 47 days after transplantation. In contrast, mice treated with 12.5, 25, and 50 mg/kg of DS-1594a·succinate survived till day 47, showing a significant survival benefit of DS-1594a (ILS: 64%, 185%, > 254%, respectively; p = 0.0018). Although mice treated with 12.5 or 25 mg/kg DS-1594a·succinate died eventually (67% and 185% ILS, respectively), all mice treated with DS-1594a·succinate 50 mg/kg remained alive 160 days after inoculation (Fig. 6D) and did not exhibit weight loss. Notably, the hCD45 positivity rate in the bone marrow at termination (day 161) for the 50 mg/kg twice-daily DS-1594a·succinate dosage group (n = 6) was ≤ 0.4%, which was similar to that in the NI group (data not shown). This finding suggested that *MLL1-r* ALL cells may have been completely eradicated from the bone marrow. Consistently, the mean spleen weight per gram of body weight was 5.71 mg in the vehicle group vs. 0.80–1.00 mg in the DS-1594a·succinate group and 1.30 mg in the NI group (data not shown).

In the pharmacokinetics (PK) evaluation, the time to reach maximum plasma concentration (Tmax) values of DS-1594a in plasma remained constant from 1.0 h to 3.0 h at the dose range from 6.25 mg/kg to 100 mg/kg. The maximum plasma concentration (Cmax) and area under the plasma concentration–time curve up to 24 h (AUC24h) values of DS-1594a increased with the dose ranging from 6.25 mg/kg to 100 mg/kg (Additional file 1: Figure S8). To investigate the hematological toxicity of DS-1594a·succinate, the white blood cell count (WBC), neutrophil count (Neutro.), red blood cell count (RBC), hemoglobin concentration (HGB), and platelet count (PLT) were measured in Crl:CD1 (ICR) mice orally administered DS-1594a·succinate (30, 100, and 300 mg/kg) for 28 days. RBC, HGB, and PLT were slightly lower in the DS-1594a·succinate-treated groups, but these effects were completely reversed after cessation of dosing even in the 300 mg/kg dose group (Additional file 1: Figure S9). No toxicologically significant findings were noted with regard to clinical signs, body weight, or food consumption or during ophthalmology, urinalysis, or necropsy evaluations at any dose level during the dosing period (data not shown). In addition, the toxic effects of DS-1594a·HCl and Ara-C on the granulocyte/macrophage colony forming unit (CFU-GM) from human cord blood-derived CD34-positive cells were evaluated by MethoCult assay. The number of CFU-GM colonies exposed to 1,000, 3,000 or 10,000 nM of DS-1594a·HCl were comparable to that of DMSO control, however, no colony was detected in the 40 or 200 nM of Ara-C treatment (Additional file 1: Figure S10). The IC_50_ for the inhibitory effect of DS-1594a·succinate on hERG channel current was 0.3 uM (data not shown), more than 100-fold higher than the GI_50_ on MV-4–11 (Fig. 1E).

## Discussion

*MLL1-r* acute leukemia patients respond poorly to currently available treatments and there is a need to develop more effective therapies directly disrupting the Menin‒MLL1 complex. Interaction of Menin with MLL1 or MLL fusion proteins is important in acute leukemias with *MLL1-r* or *NPM1c*, two gene alterations driving cancer development and growth [22, 40]. Small molecules that inhibit the Menin-MLL1 interaction provide promising therapeutic opportunities for acute leukemias with *MLL1-r* or *NPM1c*; several such small molecules, including DS-1594b, have entered clinical trials [41]. For example, thienopyrimidine compounds have been found to eradicate leukemia in a xenograft model of primary leukemic cells of the *MLL1-r* or *NPM1c* subtypes [34]. Here, we report the development and characterization of a novel, single-agent small molecule (DS-1594a) designed to target and disrupt the protein–protein interaction of Menin and MLL1 to inhibit leukemic cell growth and proliferation.

In this study, our results suggest that DS-1594a has medicinal properties distinct from those of cytarabine and that DS-1594a has the potential to be a new anticancer therapy. DS-1594a·HCl and DS-1594a·succinate displayed selective growth inhibition against AML and ALL cells with *MLL1-r* or *NPM1c* and demonstrated robust and durable antitumor activity in AML/ALL mouse models. DS-1594a·HCl selectively inhibited cellular growth of *MLL1-r* or *NPM1c* human leukemic cell lines with a GI_50_ < 30 nM, suggesting that it is a potent Menin-MLL1 inhibitor. Furthermore, DS-1594a·HCl is a potential differentiation therapeutic, as it effectively reversed differentiation blockade by reducing clonogenic potential and induced cellular differentiation in *MLL-AF9* oncogene-evoked murine AML-like cells and patient-derived *MLL1-r* leukemia cells. These effects are supported by the finding that DS-1594a·HCl and DS-1594a·succinate reduced the number of CD34 + /CD38 − cells, which indicate LIC fractions, and increased the expression of the differentiation marker CD11b. These changes were accompanied by strong inhibition of the upregulation of the expression of the MLL-fusion target genes *HOXA9, MEIS1, MEF2C*, *and PBX3*, which are important in leukemia development, cell proliferation, and self-renewal*.* RNA-seq and ChIP-seq analyses in the adult patient-derived cell line MOLM-13 and in pediatric patient-derived AML cells (NCCHD010) with *MLL1-r* showed that DS-1594a·HCl and DS-1594a·succinate concentration-dependently reduced the expression of MLL-fusion target genes and dissociated the Menin-MLL1 complex from the *MEIS1* gene locus. A significant loss of Menin binding to chromatin on Menin-MLL1–regulated genes was observed with DS-1594a·HCl or DS-1594a·succinate treatment; however, MLL1, H3K79me2, and H3K4me3 occupancy was lost on a subset of MLL-fusion target genes. The results regarding chromatin occupancy agree with a previous report wherein MLL-fusion target genes (e.g., *HOXA* and *MYB*) did not lose MLL1 or H3K79me2 occupancy [30].

The protein encoded by the *MLL-AF9* oncogene binds to the promoter regions of 139 genes [37]. GSEA-mediated gene expression profiling of MLL-AF9 target genes or *HOXA9*/*MEIS1* target genes [38, 39] indicated an association between loss of Menin on chromatin and changes in gene expression in the MOLM-13 cell line and in patient-derived primary AML cells. Moreover, a reduction in stemness with DS-1594a·succinate treatment, as assessed by LSC scores, confirmed the downregulation of stemness-associated genes, indicating suppression of LSC-related processes. These findings suggest that DS-1594a has a potential toprovide more effective therapies than current standard of care in patients with acute leukemia with *MLL1-r* and *NPM1c*.

The antitumor activity of DS-1594a·HCl and DS-1594a·succinate was reflected in the significant survival benefit in the aggressive disseminated leukemia model intravenously inoculated with MOLM-13 cells and in PDX models of *MLL1-r* or *NPM1c* acute leukemia. DS-1594a·HCl and DS-1594a·succinate completely eradicated AML and ALL cells in the MOLM-13 and NCCHD007 xenograft models, respectively, while exhibiting very low toxicity toward normal hematopoietic cells, confirming the restoration of normal myeloid cells in MOLM-13 xenografts. DS-1594a·HCl and DS-1594a·succinate resulted in complete remission (CR) in in vivo xenograft models of *MLL1-r* AML/ALL and *NPM1c* AML, suggesting that strong antitumor medicinal effects can be expected in clinical studies. The Phase I data of SNDX-5613 from AUGMENT-101 (NCT04065399) were presented at the 2021 ASH Annual Meeting [42], and SNDX-5613 exhibited promising efficacy in the *MLL1-r* AML/ALL and *NPM1c* AML. SNDX-5613 was well-tolerated, however, one of the most common adverse events was QTc prolongation (78% any grade). Our DS-1594a·succinate was optimized to overcome the issue of QTc prolongation, therefore this warrants further evaluation in clinical trial.

In this study, we evaluated the efficacy of DS-1594a monotherapy, however, it is unclear whether DS-1594a will demonstrate even stronger efficacy in combination with existing standard-of-care treatments such as Ara-C, 5-azacytidine, and venetoclax. Future studies will reveal the impact of combination therapy with DS-1594a in AML/ALL models.

## Conclusion

We developed a novel and highly potent Menin-MLL1 interaction inhibitor (DS-1594a). *MLL1-r* AML cell lines and patient-derived primary AML cells were selectively and highly sensitive to DS-1594a·HCl and DS-1594a·succinate. Compared with Ara-C, DS-1594a·HCl and DS-1594a·succinate eradicated markers of LIC fractions by enhancing differentiation and reducing colony-forming potential in *MLL1-r* AML cells in vitro. DS-1594a·HCl and DS-1594a·succinate also exhibited antitumor efficacy in MOLM-13 xenograft, *NPM1c*-mutated AML-PDX, and *MLL1-r* ALL-PDX models in vivo. Overall, our findings suggest that DS-1594a·HCl and DS-1594a·succinate may achieve CR without minimal or measurable residual disease (MRD) in individuals with *MLL1-r* AML even as single agents by inducing strong AML cell differentiation and exhibiting durable efficacy via potential LIC depletion, as MRD and LICs are major contributors to relapse in AML. In contrast, a decrease in potential LIC fractions and durable efficacy could not be confirmed for Ara-C, the current standard-of-care AML drug. Therefore, DS-1594a·HCl and DS-1594a·succinate have medicinal properties distinct from those of Ara-C and have the potential to be used as new anticancer therapies. Currently, a phase 1/2 clinical study of DS-1594a·succinate is being conducted in patients with AML and ALL with *MLL1-r* or *NPM1c* (NCT04752163).

## Supplementary Information

**Additional file 1: Figure S1.** Binding properties of DS-1594a·succinate. (A) Green sticks represent amino acid residues that form specific interactions (i.e., hydrogen bonds, π-π interactions, and CH-π interactions) with DS-1594a·succinate. (B) The magenta surface shows the solvent-accessible surface calculated from protein atoms within 5 Å from DS-1594a·succinate. DS1594a·succinate and Menin atoms are shown in yellow and green, respectively. The color scheme of the line and stick is the same as that in (A). **Figure S2.** Induction of differentiation and loss of cKit+ cells by the Menin-MLL1 inhibitor using MLL-AF9–evoked murine AML-like cells. (A) Representative microscopic images of MGG staining of MLL-AF9–evoked murine AML-like cells after 7 days of treatment with DMSO, DS-1594a·HCl (10, 20, 40 nM), or Ara-C (50, 100). (B) Based on the MGG staining images as indicated in (A), the cells were counted and classified into 5 differentiation categories (blast, myelocyte, metamyelocyte, banded neutrophil, neutrophil). (C) Representative images of FCM analysis with the indicated antibodies for MLL-AF9–evoked murine AML-like cells after 7 days of treatment with DMSO, DS-1594a·succinate (10, 20, and 40 nM), or Ara-C (50 and 100 nM). (D) RT‒qPCR was performed in MLL-AF9–evoked murine AML-like cells after treatment with DS-1594a·succinate (10, 20, 40 nM) or Ara-C (50, 100) for 7 days. The expression levels of Kit (Cd117), Itgam (Cd11b), and Ly6g were normalized to that of Actb and compared to those in the DMSO-treated controls (mean ± SD; n=3). (E) FCM analysis with the cKit antibody for MLL-AF9–evoked murine AML-like cells after 4 days of treatment with DMSO or DS-1594a·succinate (1.5, 4.6, 14 and 41 nM). The bars represent the mean ± SD; n=3. MGG, May-Grünwald-Giemsa; AML, acute myeloid leukemia; Ara-C, cytarabine; MLL, mixedlineage leukemia; APC, allophycocyanin; PE, phycoerythrin. **Figure S3.** Effect of the Menin-MLL1 inhibitor on the induction of CD11b+ differentiation and loss of CD34+/CD38- AML cells in MLL1-r+ AML patient-derived cells in vitro. Patient-derived primary AML cells (AML#8531, NCCHD010, and AML676) were treated for 7 days with DMSO, DS-1594a·succinate, or Ara-C at the indicated concentrations. Representative images of dot plots and histograms in FCM analysis with anti-human CD34/CD38 antibodies (A) and an anti-human CD11b antibody (B) are shown. (C) RT‒qPCR was performed in AML#8531 and AML676 cells after treatment with DS-1594a·succinate (30, 100, 300, nM) or Ara-C (30, 100) for 7 days. The expression levels of CD34 and ITGAM (CD11b) were normalized to that of ACTB and compared to those in the DMSO-treated controls (mean ± SD; n=3). hCD, human CD. **Figure S4.** Effect of the Menin-MLL1 inhibitor on the induction of differentiation and loss of potential LICs in NPM1c AML patient-derived cells in vitro. Patient-derived primary NPM1c AML cells (AML#7789, AML#7915, AML#7919) were treated for 7 days with the indicated compounds. (A) FCM analysis of CD33+ cells with anti-human CD34 and CD38 antibodies (AML#7789) or viable cells with anti-human CD33 and CD123 antibodies (AML#7915 and AML#7919). (B) FCM analysis of viable cells with an anti-human CD11b antibody. (C) RTqPCR to detect the expression levels of MEIS1 and HOXA9 (mean ± SD; n = 2 [technical replicates]) normalized to those of GAPDH. AML, acute myeloid leukemia; Ara-C, cytarabine; FCM, flow cytometry; hCD, human CD; LIC, leukemia-initiating cell; NPM1c, mutated nucleophosmin 1; PE, phycoerythrin; PerCP, peridinin-chlorophyll protein complex; RT‒qPCR, real-time quantitative PCR. **Figure S5.** Changes in Menin/MLL1 occupancies in NCCHD010 cells. (A) RNA-seq was performed in MOLM-13 and NCCHD010 cells treated with DS-1594a·succinate for 3 and 7 days, respectively, at the indicated concentrations. The graphs represent the expression levels of the indicated genes compared to those in the DMSO-treated controls (mean ± SD, n=3). (B) ChIP-seq was performed in NCCHD010 cells treated with 500 nM or 5 μM DS-1594a·succinate for 3 days. Normalized coverage tracks of menin, MLL1, H3K79me2, and H3K4me3 ChIP-seq signals (read per million) at selected target genes in NCCHD010 are shown. The peaks around the TSS are shown. **Figure S6.** Tumor burden in an AML-PDX model (AM7577) treated with the Menin-MLL1 inhibitor. The tumor burdens in the PB, spleens, and BM of AM7577 mice were assessed by the presence of hCD45+ cells at termination (day 35; n = 6 mice per group). AML, acute myeloid leukemia; hCD, human CD; PB, peripheral blood; PDX, patientderived xenograft. **Figure S7.** In vivo antitumor effect of the Menin-MLL1 inhibitor in the ALL-PDX model (NCCHD007). RTqPCR to assess the mRNA expression levels of human MEIS1, MEF2C, and CDK6 in the bone marrow of NCCHD007 mice 1 day after 28 days of treatment (day 38) with DS1594a·succinate. The expression levels were normalized to human GAPDH mRNA levels (mean ± SD, n = 3). **Figure S8.** Pharmacokinetics Parameters in Plasma after Single Oral Administration of DS-1594a·succinate to mice. Blood samples were collected at 1, 2, 6, and 24 hours after a single oral administration of DS-1594a·succinate to severe combined immunodeficiency (SCID) mice at 6.25, 12.5, 25, 50, and 100 mg/kg. Values are mean ± standard deviation (n = 3/group). AUC24h, area under the plasma concentration-time curve up to 24 hours; Cmax, maximum plasma concentration; Tmax, time to reach maximum plasma concentration. **Figure S9.** Hematology in mice treated orally with DS-1594a·succinate for 28 days before a 28-day recovery period. White blood cell count (WBC), neutrophil count (Neutro.), red blood cell count (RBC), hemoglobin concentration (HGB), and platelet count (PLT) were measured in Crl:CD1 (ICR) mice that were orally administered 0 (vehicle control), 30, 100, or 300 mg/kg (DS-1594a·succinate) once daily for 28 consecutive days (end of the dosing period, 10 mice/group). Five animals treated at dose levels of 0 and 300 mg/kg were used to evaluate the reversibility of toxicity during a 28-day recovery period (end of the dosing recovery period, 5 mice/group). **Figure S10.** The number of the granulocyte/macrophage colony-forming unit (CFU-GM) of CB-CD34+ using MethoCult H4535. The total number of CFU-GM from human cord blood-derived CD34+ cells was counted using microscopy at 12 days post seeding in MethoCult H4535 and treatment with DMSO, DS-1594·HCl or Ara-C. Each circle indicates the CFU-GM number of each plate and bars represent mean value, n = 2. **Table S1.** Statistics for data collection and phase refinement. **Table S2.** Antibodies used for FCM in murine MA9 cells. **Table S3.** Sample types used for FCM in murine MA9 cells. **Table S4.** Antibodies used for ChIP. **Table S5.** Antibodies used in FCM analysis of human-derived AML676 and NCCHD010 cells. **Table S6.** Antibodies used in FCM analysis of human-derived AML#8531, AML#7789, AML#7915, and AML#7919 cells. **Table S7.** Information on primary patient samples.

**Additional file 2.** Original raw WB data for Fig. 1D.

## Data Availability

All data generated or analyzed during this study are included in this published article and its supplementary information files.
