# Supplementary material for: A novel Menin-MLL1 inhibitor, DS-1594a, prevents the progression of acute leukemia with rearranged MLL1 or mutated NPM1
Source: Cancer Cell Int. 2023 Feb 25;23:36. doi: 10.1186/s12935-023-02877-y (PMC9960487; doi:10.1186/s12935-023-02877-y)
Supplement: Supplementary file 1 — Additional file 1: Figure S1. Binding properties of DS-1594a·succinate. (A) Green sticks represent amino acid residues that form specific interactions (i.e., hydrogen bonds, π-π interactions, and CH-π interactions) with DS-1594a·succinate. (B) The magenta surface shows the solvent-accessible surface calculated from protein atoms within 5 Å from DS-1594a·succinate. DS1594a·succinate and Menin atoms are shown in yellow and green, respectively. The color scheme of the line and stick is the same as that in (A). Figure S2. Induction of differentiation and loss of cKit+ cells by the Menin-MLL1 inhibitor using MLL-AF9–evoked murine AML-like cells. (A) Representative microscopic images of MGG staining of MLL-AF9–evoked murine AML-like cells after 7 days of treatment with DMSO, DS-1594a·HCl (10, 20, 40 nM), or Ara-C (50, 100). (B) Based on the MGG staining images as indicated in (A), the cells were counted and classified into 5 differentiation categories (blast, myelocyte, metamyelocyte, banded neutrophil, neutrophil). (C) Representative images of FCM analysis with the indicated antibodies for MLL-AF9–evoked murine AML-like cells after 7 days of treatment with DMSO, DS-1594a·succinate (10, 20, and 40 nM), or Ara-C (50 and 100 nM). (D) RT‒qPCR was performed in MLL-AF9–evoked murine AML-like cells after treatment with DS-1594a·succinate (10, 20, 40 nM) or Ara-C (50, 100) for 7 days. The expression levels of Kit (Cd117), Itgam (Cd11b), and Ly6g were normalized to that of Actb and compared to those in the DMSO-treated controls (mean ± SD; n=3). (E) FCM analysis with the cKit antibody for MLL-AF9–evoked murine AML-like cells after 4 days of treatment with DMSO or DS-1594a·succinate (1.5, 4.6, 14 and 41 nM). The bars represent the mean ± SD; n=3. MGG, May-Grünwald-Giemsa; AML, acute myeloid leukemia; Ara-C, cytarabine; MLL, mixedlineage leukemia; APC, allophycocyanin; PE, phycoerythrin. Figure S3. Effect of the Menin-MLL1 inhibitor on the induction of CD11b+ differentiation and lo [file 12935_2023_2877_MOESM1_ESM.docx]

**Additional file Information**

**Additional file Results**


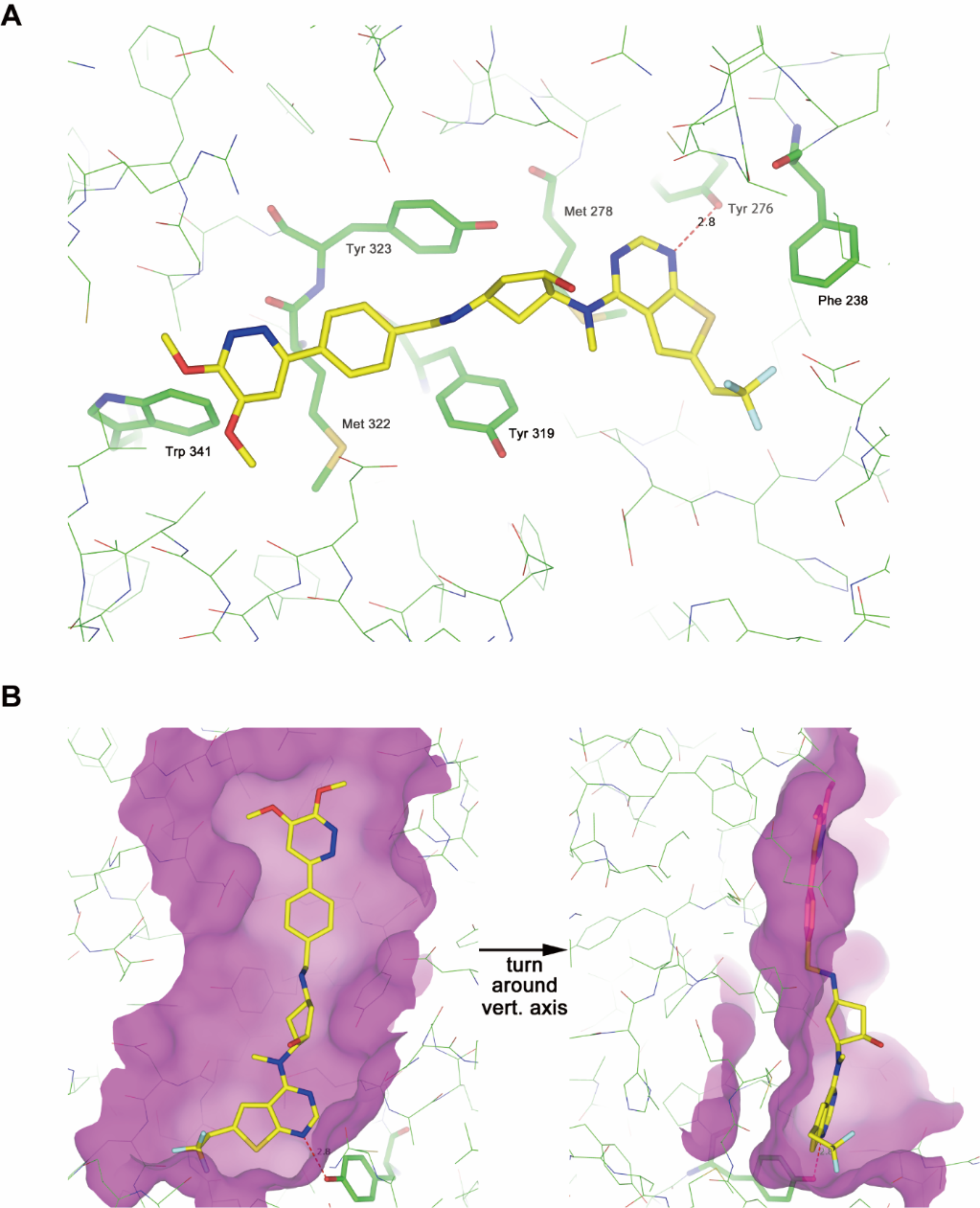


**Additional file 1: Figure S1. Binding properties of DS-1594a·succinate.** (**A**) Green sticks represent amino acid residues that form specific interactions (i.e., hydrogen bonds, π-π interactions, and CH-π interactions) with DS-1594a·succinate. (**B**) The magenta surface shows the solvent-accessible surface calculated from protein atoms within 5 Å from DS-1594a·succinate. DS‑1594a·succinate and Menin atoms are shown in yellow and green, respectively. The color scheme of the line and stick is the same as that in (A).

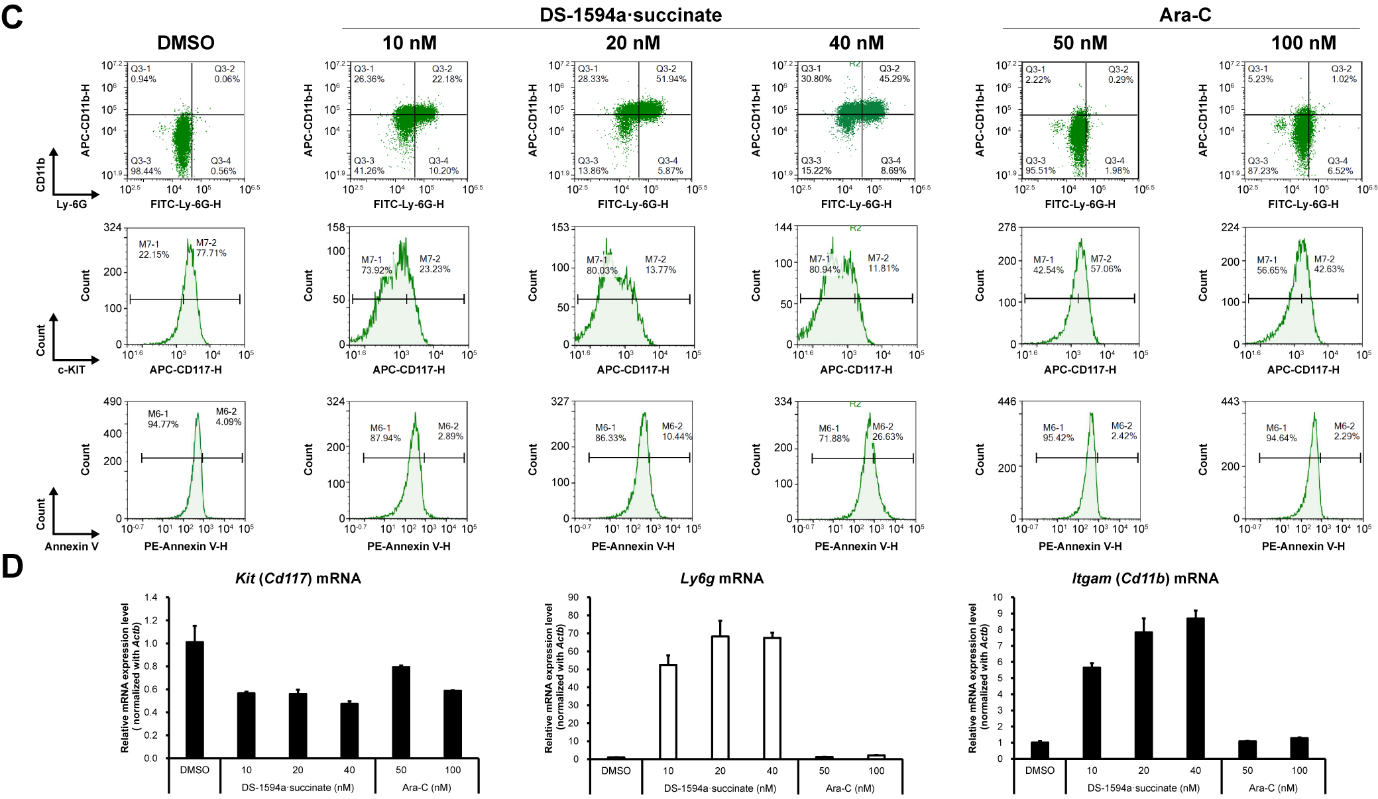


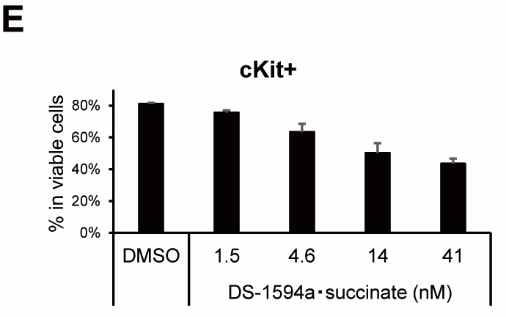


**Additional file 1: Figure S2. Induction of differentiation and loss of cKit+ cells by the Menin-MLL1 inhibitor using *MLL-AF9*–evoked murine AML-like cells.** (**A**) Representative microscopic images of MGG staining of *MLL-AF9*–evoked murine AML-like cells after 7 days of treatment with DMSO, DS-1594a·HCl (10, 20, 40 nM), or Ara-C (50, 100). (**B**) Based on the MGG staining images as indicated in (A), the cells were counted and classified into 5 differentiation categories (blast, myelocyte, metamyelocyte, banded neutrophil, neutrophil). (**C**) Representative images of FCM analysis with the indicated antibodies for *MLL-AF9*–evoked murine AML-like cells after 7 days of treatment with DMSO, DS-1594a·succinate (10, 20, and 40 nM), or Ara-C (50 and 100 nM). (**D**) RT‒qPCR was performed in *MLL-AF9*–evoked murine AML-like cells after treatment with DS-1594a·succinate (10, 20, 40 nM) or Ara-C (50, 100) for 7 days. The expression levels of *Kit* (*Cd117*), *Itgam* (*Cd11b*), and *Ly6g* were normalized to that of *Actb* and compared to those in the DMSO-treated controls (mean ± SD; n=3). (**E**) FCM analysis with the cKit antibody for *MLL-AF9–*evoked murine AML-like cells after 4 days of treatment with DMSO or DS-1594a·succinate (1.5, 4.6, 14 and 41 nM). The bars represent the mean ± SD; n=3.

MGG, May-Grünwald-Giemsa; AML, acute myeloid leukemia; Ara-C, cytarabine; MLL, mixed‑lineage leukemia; APC, allophycocyanin; PE, phycoerythrin.


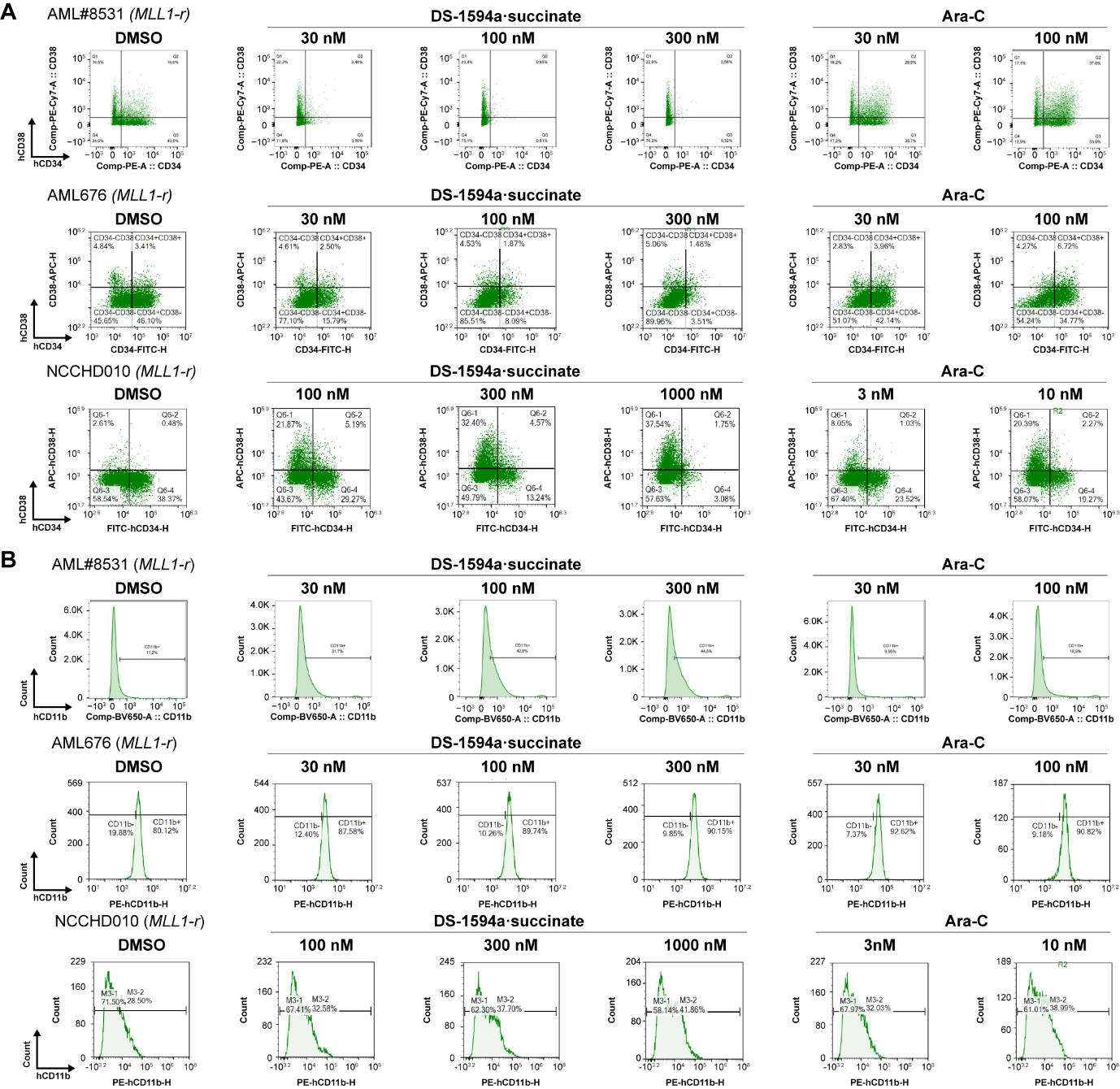


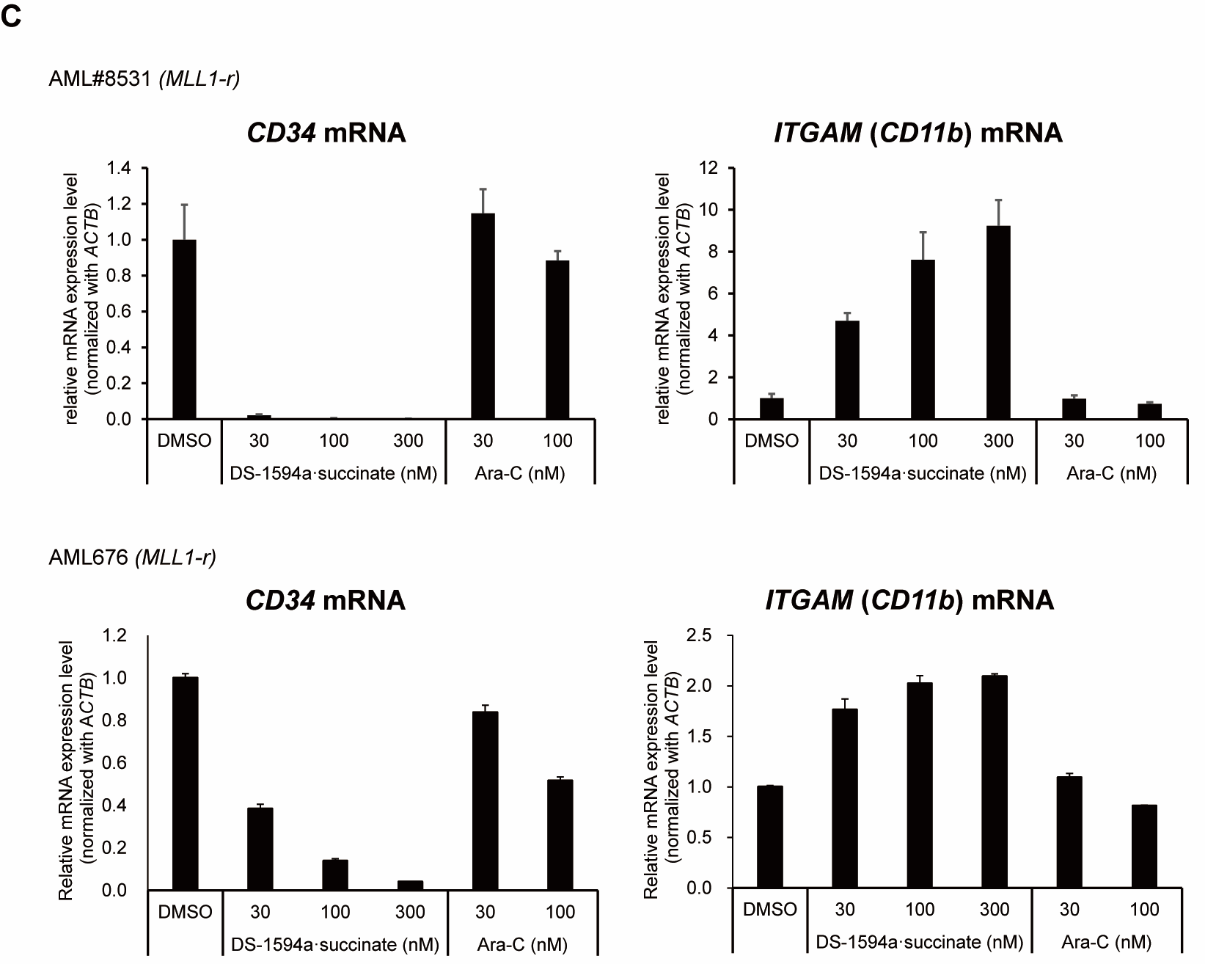


**Additional file 1: Figure S3.** **Effect of the Menin-MLL1 inhibitor on the induction of CD11b+ differentiation and loss of CD34+/CD38- AML cells in *MLL1-r*+ AML patient-derived cells in vitro.**

Patient-derived primary AML cells (AML#8531, NCCHD010, and AML676) were treated for 7 days with DMSO, DS-1594a·succinate, or Ara-C at the indicated concentrations. Representative images of dot plots and histograms in FCM analysis with anti-human CD34/CD38 antibodies (**A**) and an anti-human CD11b antibody (**B**) are shown. (**C**) RT‒qPCR was performed in AML#8531 and AML676 cells after treatment with DS-1594a·succinate (30, 100, 300, nM) or Ara-C (30, 100) for 7 days. The expression levels of *CD34* and *ITGAM* (*CD11b*) were normalized to that of *ACTB* and compared to those in the DMSO-treated controls (mean ± SD; n=3). hCD, human CD.

**Additional file 1: Figure S4.** **Effect of the Menin-MLL1 inhibitor on the induction of differentiation and loss of potential LICs in *NPM1c* AML patient-derived cells in vitro.** Patient-derived primary *NPM1c* AML cells (AML#7789, AML#7915, AML#7919) were treated for 7 days with the indicated compounds. (**A**) FCM analysis of CD33+ cells with anti-human CD34 and CD38 antibodies (AML#7789) or viable cells with anti-human CD33 and CD123 antibodies (AML#7915 and AML#7919). (**B**) FCM analysis of viable cells with an anti-human CD11b antibody. (**C**) RT‑qPCR to detect the expression levels of *MEIS1* and *HOXA9* (mean ± SD; n = 2 [technical replicates]) normalized to those of *GAPDH.*

AML, acute myeloid leukemia; Ara-C, cytarabine; FCM, flow cytometry; hCD, human CD; LIC, leukemia-initiating cell; *NPM1c*, mutated *nucleophosmin 1*; PE, phycoerythrin; PerCP, peridinin-chlorophyll protein complex; RT‒qPCR, real-time quantitative PCR.


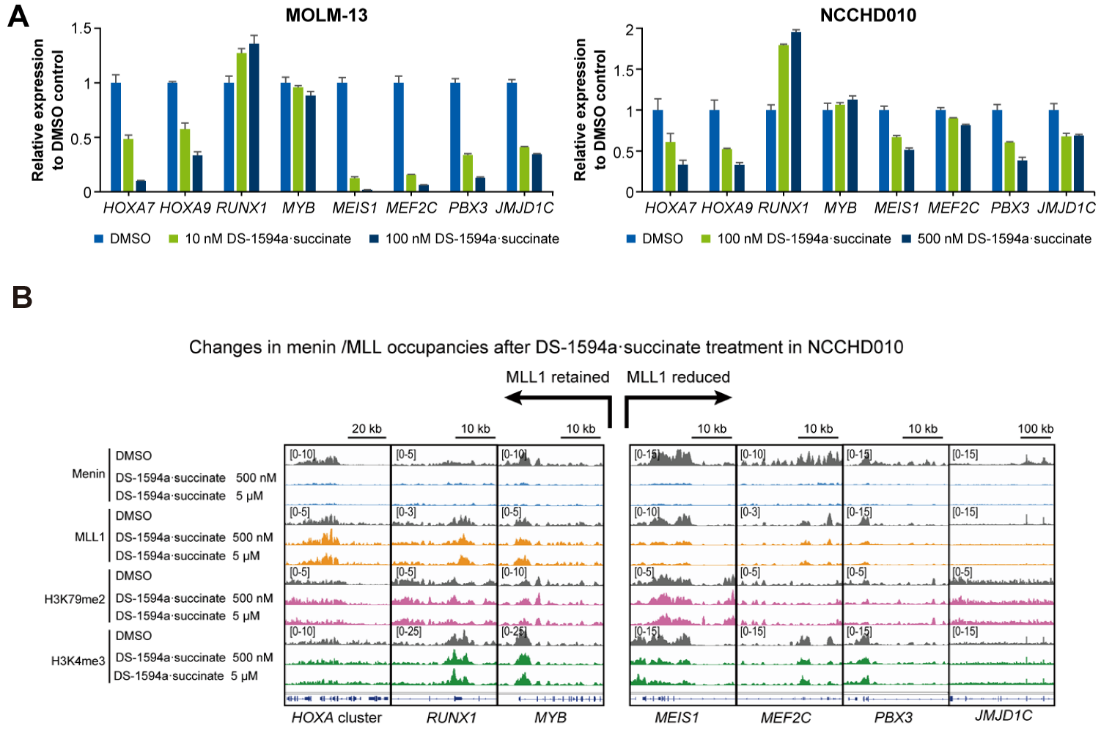


**Additional file 1: Figure S5. Changes in Menin/MLL1 occupancies in NCCHD010 cells.**

(**A**) RNA-seq was performed in MOLM-13 and NCCHD010 cells treated with DS-1594a·succinate for 3 and 7 days, respectively, at the indicated concentrations. The graphs represent the expression levels of the indicated genes compared to those in the DMSO-treated controls (mean ± SD, n=3). (**B**) ChIP-seq was performed in NCCHD010 cells treated with 500 nM or 5 μM DS-1594a·succinate for 3 days. Normalized coverage tracks of menin, MLL1, H3K79me2, and H3K4me3 ChIP-seq signals (read per million) at selected target genes in NCCHD010 are shown. The peaks around the TSS are shown.

MLL1, mixed-lineage leukemia 1; TSS, transcription start site.

**Additional file 1: Figure S6. Tumor burden in an AML-PDX model (AM7577) treated with the Menin-MLL1 inhibitor.** The tumor burdens in the PB, spleens, and BM of AM7577 mice were assessed by the presence of hCD45+ cells at termination (day 35; n = 6 mice per group).

AML, acute myeloid leukemia; hCD, human CD; PB, peripheral blood; PDX, patient‑derived xenograft.

**Additional file 1: Figure S7. In vivo antitumor effect of the Menin-MLL1 inhibitor in the ALL-PDX model (NCCHD007).** RT‑qPCR to assess the mRNA expression levels of human *MEIS1*, *MEF2C*, and *CDK6* in the bone marrow of NCCHD007 mice 1 day after 28 days of treatment (day 38) with DS‑1594a·succinate. The expression levels were normalized to human *GAPDH* mRNA levels (mean ± SD, n = 3).

ALL, acute lymphoblastic leukemia; CTL, control (vehicle); PDX, patient‑derived xenograft; RT‑qPCR, real-time quantitative PCR.


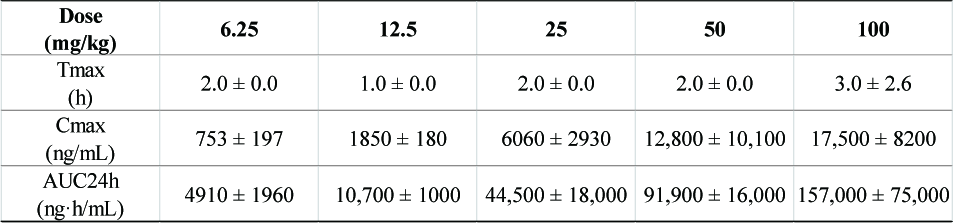


**Additional file 1: Figure S8. Pharmacokinetics Parameters in Plasma after Single Oral Administration of DS-1594a·succinate to mice.** Blood samples were collected at 1, 2, 6, and 24 hours after a single oral administration of DS-1594a·succinate to severe combined immunodeficiency (SCID) mice at 6.25, 12.5, 25, 50, and 100 mg/kg. Values are mean ± standard deviation (n = 3/group). AUC24h, area under the plasma concentration-time curve up to 24 hours; Cmax, maximum plasma concentration; Tmax, time to reach maximum plasma concentration.


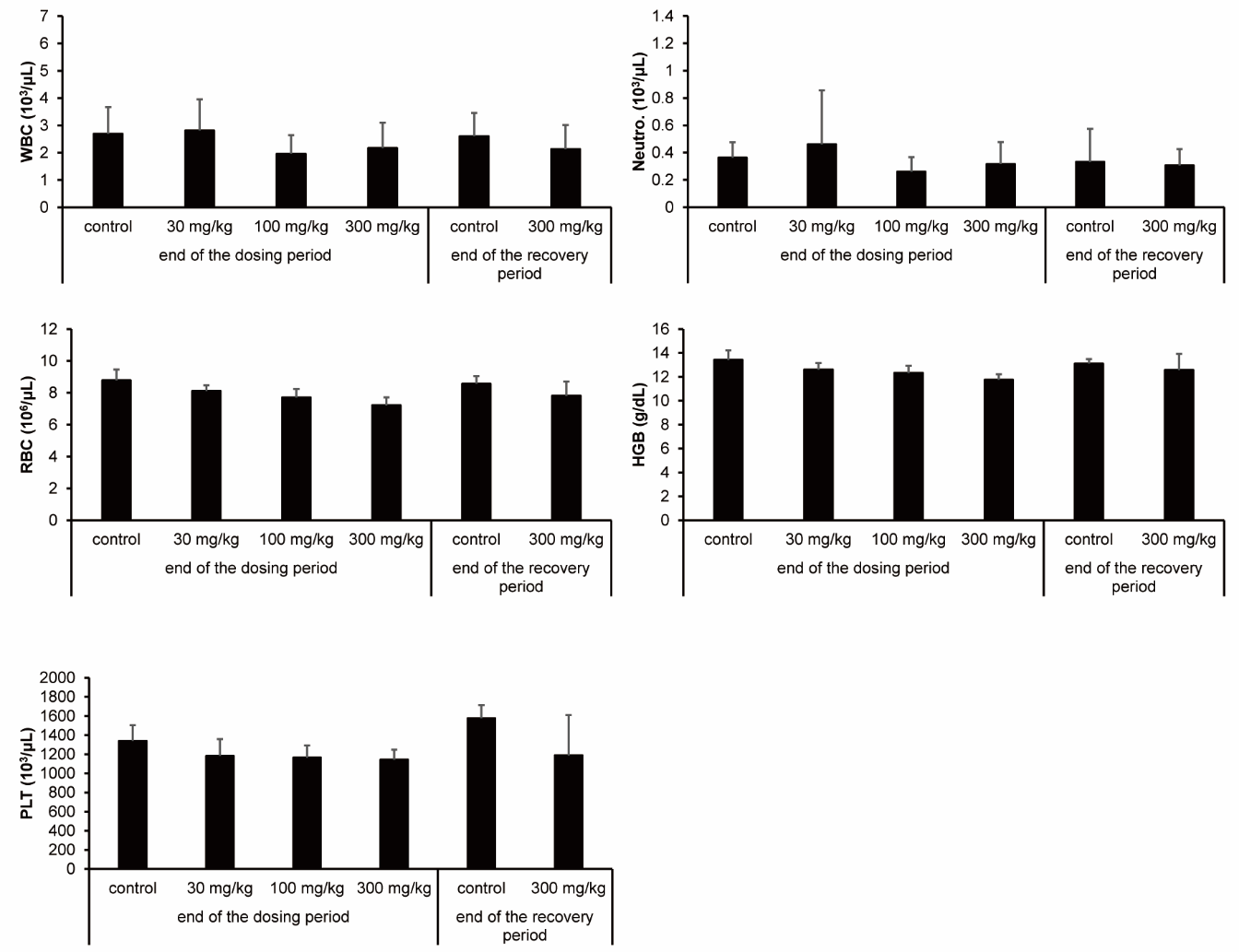


**Additional file 1: Figure S9. Hematology in mice treated orally with DS-1594a·succinate for 28 days before a 28-day recovery period.** White blood cell count (WBC), neutrophil count (Neutro.), red blood cell count (RBC), hemoglobin concentration (HGB), and platelet count (PLT) were measured in Crl:CD1 (ICR) mice that were orally administered 0 (vehicle control), 30, 100, or 300 mg/kg (DS-1594a·succinate) once daily for 28 consecutive days (end of the dosing period, 10 mice/group). Five animals treated at dose levels of 0 and 300 mg/kg were used to evaluate the reversibility of toxicity during a 28-day recovery period (end of the dosing recovery period, 5 mice/group).

**Additional file 1: Figure S10. The number of the granulocyte/macrophage colony-forming unit (CFU-GM) of CB-CD34+ using MethoCult H4535.** The total number of CFU-GM from human cord blood-derived CD34+ cells was counted using microscopy at 12 days post seeding in MethoCult H4535 and treatment with DMSO, DS-1594·HCl or Ara-C. Each circle indicates the CFU-GM number of each plate and bars represent mean value, n = 2.

**Additional file 1: *Methods***

**Chemical synthesis of DS-1594a, DS-1594a·HCl, and DS-1594a·succinate (DS-1594b)**

**General information**

All solvents and reagents were used as acquired from commercial sources without purification. Silica gel or amino-silica gel column chromatography was performed on commercially available column cartridges and a Biotage SP1, a Yamazen YFLC-W-Prep2XY, or a Shoko scientific Purif-espoir2 purification system. TLC was performed using Merck TLC plates pre-coated with silica gel 60 F_254_, 60 NH_2_ F_254_s and Wako with NH_2_ silica gel NH_2_ F_254_. Proton NMR (^1^H-NMR) spectra in deuterated chloroform (CDCl_3_) or deuterated dimethylsulfoxide (DMSO-D_6_) were recorded on a JEOL JNM-ECX400P spectrometer and a JEOL JNM-ECS400 spectrometer. Multiplicity abbreviations are s = singlet, d = doublet, m = multiplet, dd = doublet of doublets, and br = broad. ^1^H-NMR chemical shifts are given in ppm (δ) from tetramethylsilane, which was used as the internal standard. Mass spectra were measured using an Agilent G6130B (APCI) spectrometer.

**Preparation of methyl (1*R*,4*S*)-4-aminocyclopent-2-ene-1-carboxylate hydrochloride**

To a stirred solution of (1*S*)-(+)-2-azabicyclo[2.2.1]hept-5-en-3-one (98.6 g, 885 mmol) in methanol (300 mL), thionyl chloride (40 mL, 531 mmol) was added over 50 min at 0°C. The reaction mixture was stirred at 0°C for 2 h and concentrated under reduced pressure. The obtained solid was suspended in ethyl acetate and collected by filtration to give methyl (1*R*,4*S*)-4-aminocyclopent-2-ene-1-carboxylate hydrochloride (158 g, 100%) as a colorless solid. ^1^H-NMR (DMSO-D_6_) δ: 1.88-1.99 (1H, m), 2.48-2.62 (1H, m), 3.64-3.74 (4H, m), 4.15-4.23 (1H, m), 5.84-5.90 (1H, m), 6.07-6.11 (1H, m), 8.20 (3H, br s).

**Preparation of methyl (1*R*,4*S*)-4-[(*tert*-butoxycarbonyl)amino]cyclopent-2-ene-1-carboxylate**

To a stirred solution of methyl (1*R*,4*S*)-4-aminocyclopent-2-ene-1-carboxylate hydrochloride (158 g, 888 mmol) in tetrahydrofuran (700 mL) and water (280 mL), di-tert-butyl dicarbonate (194 g, 888 mmol) and sodium carbonate (104 g, 977 mmol) were added at 0°C, and the mixture was stirred overnight at room temperature. Water was added to the reaction solution, and the mixture was extracted with ethyl acetate. The organic layer was washed with saturated brine, dried over anhydrous sodium sulfate, and concentrated under reduced pressure to give methyl (1*R*,4*S*)-4-[(tert-butoxycarbonyl)amino]cyclopent-2-ene-1-carboxylate (214 g, 100%) as a colorless oil. ^1^H-NMR (CDCl_3_) δ: 1.44 (9H, s), 1.82-1.91 (1H, m), 2.46-2.57 (1H, m), 3.45-3.52 (1H, m), 3.71 (3H, s), 4.75-4.84 (1H, m), 4.86-4.96 (1H, m), 5.84-5.91 (2H, m). MS (m/z): 142 (M-Boc+H)^+^.

**Preparation of methyl (3a*S*, 5*S*, 6*S*, 6a*S*)-6-bromo-2-oxohexahydro-2*H*-cyclopent[*d*] [1,3]oxazole-5-carboxylate**

To a stirred solution of methyl (1*R*,4*S*)-4-[(tert-butoxycarbonyl)amino]cyclopent-2-ene-1-carboxylate (214 g, 888 mmol) in tetrahydrofuran (700 mL) and water (70 mL), *N*-bromosuccinimide (174 g, 977 mmol) was added at 0°C, and the mixture was stirred overnight at room temperature. The reaction mixture was concentrated under reduced pressure, and ethyl acetate was added thereto. The organic layer was washed with 1 N hydrochloric acid, saturated aqueous sodium hydrogencarbonate solution, saturated aqueous sodium thiosulfate solution, and saturated brine; dried over anhydrous sodium sulfate; and concentrated under reduced pressure. The residue was suspended in ethyl acetate/n-hexane, and the solid was collected by filtration. The obtained solid was resuspended in ethyl acetate/n-hexane and collected by filtration to give methyl (3a*S*, 5*S*, 6*S*, 6a*S*)-6-bromo-2-oxohexahydro-2*H*-cyclopent[*d*] [1,3]oxazole-5-carboxylate as a colorless solid. The filtrate was concentrated under reduced pressure, the residue was suspended in ethyl acetate/n‑hexane, and the solid was collected by filtration. Ethyl acetate/n-hexane and water were added to the obtained solid, and the mixture was subjected to liquid separation. The organic layer was dried over anhydrous sodium sulfate and concentrated under reduced pressure to give a colorless solid as the second crop. In total, methyl (3a*S*, 5*S*, 6*S*, 6a*S*)-6-bromo-2-oxohexahydro-*2H*-cyclopent[*d*] [1,3]oxazole-5-carboxylate (124 g) was obtained at a 53% yield.^1^H-NMR (CDCl_3_) δ: 2.39-2.47 (1H, m), 2.48-2.55 (1H, m), 3.21-3.25 (1H, m), 3.76 (3H, s), 4.42-4.45 (1H, m), 4.79 (1H, s), 5.16 (1H, dd, J = 7.6, 1.5 Hz), 5.95 (1H, br s). MS (m/z): 264, 266 (M+H)^+^.

**Preparation of (3*R*,4*S*)-4-[(*tert*-butoxycarbonyl)amino]-3-hydroxycyclopent-1-ene-1-carboxylic acid**

To a stirred solution of methyl (3a*S*, 5*S*, 6*S*, 6a*S*)-6-bromo-2-oxohexahydro-2*H*-cyclopent[*d*] [1,3]oxazole-5-carboxylate (124 g, 470 mmol) in methanol (580 mL) and water (580 mL), potassium hydroxide (106 g, 1.88 mol) was added at 0°C, and the mixture was stirred at 90°C for 18 h. The reaction mixture was concentrated under reduced pressure, tetrahydrofuran (180 mL) and di-tert-butyl dicarbonate (102 g) were added thereto at 0°C, and the mixture was stirred at room temperature for 4 h. The mixture was neutralized (pH = 4) with 5 N hydrochloric acid and 2 N hydrochloric acid at 0°C and extracted 5 times with ethyl acetate. The combined organic layers were dried over anhydrous sodium sulfate, and the solvent was evaporated under reduced pressure. The obtained solid was suspended in ethyl acetate/n‑hexane, collected by filtration, and dried under reduced pressure to give (3*R*,4*S*)-4-[(tert-butoxycarbonyl)amino]-3-hydroxycyclopent-1-ene-1-carboxylic acid as a colorless solid. The filtrate was concentrated, and the obtained solid was suspended in ethyl acetate/n‑hexane, collected by filtration, and dried under reduced pressure to give a colorless solid as second crop. In total, (3*R*,4*S*)-4-[(tert-butoxycarbonyl)amino]-3-hydroxycyclopent-1-ene-1-carboxylic acid (96.9 g) was obtained at an 85% yield.. ^1^H-NMR (DMSO-D_6_) δ: 1.39 (9H, s), 2.30-2.45 (1H, m), 2.53-2.63 (1H, m), 3.91-4.04 (1H, m), 4.47-4.54 (1H, m), 4.97-5.12 (1H, m), 6.35 (1H, d, J = 7.9 Hz), 6.52 (1H, d, J = 1.8 Hz), 12.53 (1H, br s). MS (m/z): 242 (M-H)^-^.

**Preparation of (3*S*, 4*R*)-3-[(*tert*-butoxycarbonyl)amino]-4-hydroxycyclopentane-1-carboxylic acid (diastereomer mixture)**

A mixture of (3*R*,4*S*)-4-[(tert-butoxycarbonyl)amino]-3-hydroxycyclopent-1-ene-1-carboxylic acid (96.9 g, 398 mmol), 10% palladium on carbon wet [50% wet (w/w), 10.4 g] in methanol (780 mL) was stirred under hydrogen atmosphere for 7.5 h. The reaction solution was filtered through the celite pad, and the filtrate was concentrated under reduced pressure to give (3*S*, 4*R*)-3-[(*tert*-butoxycarbonyl)amino]-4-hydroxycyclopentane-1-carboxylic acid (105 g, quant.) as a colorless solid. ^1^H-NMR (DMSO-D_6_) δ: 1.38 (9H, s), 1.69-2.08 (4H, m), 2.59-2.72 (1H, m), 3.52-3.73 (1H, m), 3.84-3.99 (1H, m), 6.15-6.29 (1H, m). MS (m/z): 146 (M-Boc+H)^+^.

**Preparation of (1*R*, 3*S*, 4*R*)-3-[(*tert*-butoxycarbonyl)amino]-4-hydroxycyclopentane-1-carboxylic acid 2-methylpropan-2-amine salt**

A mixture of (3*S*, 4*R*)-3-[(*tert*-butoxycarbonyl)amino]-4-hydroxycyclopentane-1-carboxylic acid (105 g, 398 mmol), tert-butylamine (42.5 mL, 400 mmol) in methanol (90 mL) and tert‑butyl methyl ether (675 mL) was allowed to stand in the refrigerator for 3 days. The precipitated solid was collected by filtration and dried under reduced pressure to give (1*R*, 3*S*, 4*R*)-3-[(*tert*-butoxycarbonyl)amino]-4-hydroxycyclopentane-1-carboxylic acid 2-methylpropan-2-amine salt (108 g, 81%) as a colorless solid. ^1^H-NMR (DMSO-D_6_) δ: 1.18 (9H, s), 1.35 (9H, s), 1.49-1.62 (1H, m), 1.64-1.72 (1H, m), 1.72-1.85 (1H, m), 1.95-2.06 (1H, m), 2.44-2.53 (1H, m), 3.51-3.62 (1H, m), 3.71-3.79 (1H, m), 6.13 (1H, d, J = 7.9 Hz). MS (m/z): 146 (M-Boc+H)^+^.

**Preparation of (1*R*, 3*S*, 4*R*)-3-[(*tert*-butoxycarbonyl)amino]-4-hydroxycyclopentane-1-carboxylic acid**

A solution of (1*R*, 3*S*, 4*R*)-3-[(*tert*-butoxycarbonyl)amino]-4-hydroxycyclopentane-1-carboxylic acid 2-methylpropan-2-amine salt (108 g, 339 mmol) in water (200 mL) was adjusted to pH 3 with 2 N hydrochloric acid (150 mL) at 0°C. The mixture was extracted thrice with dichloromethane:methanol (10:1); the combined organic layers were dried over anhydrous sodium sulfate and concentrated under reduced pressure to give (1*R*, 3*S*, 4*R*)-3-[(*tert*-butoxycarbonyl)amino]-4-hydroxycyclopentane-1-carboxylic acid (63.9 g, 77%) as a pale brown solid. ^1^H-NMR (DMSO-D_6_) δ: 1.38 (9H, s), 1.70-1.84 (2H, m), 1.85-1.98 (1H, m), 1.98-2.08 (1H, m), 2.61-2.71 (1H, m), 3.51-3.64 (1H, m), 3.84-3.92 (1H, m), 6.21 (1H, d, J = 7.9 Hz). MS (m/z): 146 (M-Boc+H)^+^.

**Preparation of methoxymethyl (1*R*, 3*S*, 4*R*)-3-[(*tert*-butoxycarbonyl)amino]-4-(methoxymethoxy)cyclopentane-1-carboxylate**

To a stirred solution of (1*R*, 3*S*, 4*R*)-3-[(*tert*-butoxycarbonyl)amino]-4-hydroxycyclopentane-1-carboxylic acid (61.6 g) in 1,2-dimethoxyethane (1000 mL), chloromethyl methyl ether (56.7 mL, 753 mmol), *N*,*N*-diisopropylethylamine (262 mL, 1510 mmol), and sodium iodide (75.2 g, 502 mmol) were added, and the mixture was heated under the reflux for 1 h. The reaction solution was allowed to cool to room temperature, saturated brine and water were added thereto, and the mixture was extracted twice with ethyl acetate. The combined organic layers were dried over anhydrous sodium sulfate and concentrated under reduced pressure. The residue was purified by silica gel column chromatography (n-hexane/ethyl acetate) to give methoxymethyl (1*R*, 3*S*, 4*R*)-3-[(*tert*-butoxycarbonyl)amino]-4-(methoxymethoxy)cyclopentane-1-carboxylate (66.5 g, 80%) as a pale brown oil. ^1^H-NMR (CDCl_3_) δ: 1.44 (9H, s), 1.87-1.98 (1H, m), 2.04-2.17 (1H, m), 2.17-2.27 (1H, m), 2.27-2.38 (1H, m), 2.81-2.92 (1H, m), 3.37 (3H, s), 3.46 (3H, s), 3.92-4.07 (2H, m), 4.61 (1H, d, J = 6.7 Hz), 4.70 (1H, d, J = 6.7 Hz), 5.09 (1H, d, J = 10.0 Hz), 5.24 (2H, s). MS (m/z): 234 (M‑Boc+H)^+^.

**Preparation of (1*R*, 3*S*, 4*R*)-3-[(*tert*-butoxycarbonyl)amino]-4-(methoxymethoxy)cyclopentane-1-carboxylic acid**

To a stirred solution of methoxymethyl (1*R*, 3*S*, 4*R*)-3-[(*tert*-butoxycarbonyl)amino]-4-(methoxymethoxy)cyclopentane-1-carboxylate (66.5 g, 199 mmol) in methanol (20 mL) and tetrahydrofuran (580 mL), 1 N aqueous sodium hydroxide solution (400 mL, 400 mmol) was added, and the mixture was stirred at room temperature for 2 h. 1N hydrochloric acid (555 mL, 555 mmol) was added to the reaction solution at 0°C, and the mixture was extracted twice with ethyl acetate. The combined organic layers were washed with saturated brine, dried over anhydrous sodium sulfate, and concentrated under reduced pressure. The residue was purified by silica gel column chromatography (n-hexane/ethyl acetate) to give (1*R*, 3*S*, 4*R*)-3-[(*tert*-butoxycarbonyl)amino]-4-(methoxymethoxy)cyclopentane-1-carboxylic acid (53.6 g, 93%) as a pale yellow oil. ^1^H-NMR (CDCl_3_) δ: 1.44 (9H, s), 1.86-1.99 (1H, m), 2.03‑2.17 (1H, m), 2.17-2.26 (1H, m), 2.26-2.38 (1H, m), 2.81-2.93 (1H, m), 3.38 (3H, s), 3.91-4.08 (2H, m), 4.62 (1H, d, J = 6.7 Hz), 4.70 (1H, d, J = 6.7 Hz), 5.09 (1H, d, J = 6.7 Hz). MS (m/z): 288 (M-H)^-^.

**Preparation of benzyl *tert*-butyl [(1*R*, 3*S*, 4*R*)-4-(methoxymethoxy)cyclopentane-1,3-diyl]biscarbamate**

To a solution of (1*R*, 3*S*, 4*R*)-3-[(*tert*-butoxycarbonyl)amino]-4-(methoxymethoxy)cyclopentane-1-carboxylic acid (53.6 g, 185 mmol) in toluene (730 mL), diphenylphosphoryl azide (51.9 mL, 241 mmol) and triethylamine (33.4 mL, 241 mmol) were added, and the mixture was stirred at 90°C for 30 min. Benzyl alcohol (38.3 mL) was added to the reaction solution, and the mixture was stirred at 90°C for 2.5 h. The reaction solution was allowed to cool to room temperature, water was added, and the mixture was extracted twice with ethyl acetate. The combined organic layers were washed with saturated brine, dried over anhydrous sodium sulfate, and concentrated under reduced pressure. The residue was purified by silica gel column chromatography (n-hexane/ethyl acetate) to give benzyl *tert*-butyl [(1*R*, 3*S*, 4*R*)-4-(methoxymethoxy)cyclopentane-1,3-diyl]biscarbamate (63.2 g, 87%) as pale yellow oil. ^1^H-NMR (CDCl_3_) δ: 1.35-1.50 (10H, m), 1.70-1.78 (1H, m), 2.01‑2.19 (1H, m), 2.47-2.63 (1H, m), 3.36 (3H, s), 3.80-4.06 (2H, m), 4.08-4.24 (1H, m), 4.62 (1H, d, J = 6.7 Hz), 4.68 (1H, d, J = 6.7 Hz), 4.97-5.19 (4H, m), 7.29-7.42 (5H, m). MS (m/z): 295 (M-Boc+H)^+^.

**Preparation of benzyl [(1*R*, 3*S*, 4*R*)-3-amino-4-hydroxycyclopentyl]carbamate hydrochloride**

To a solution of benzyl *tert*-butyl [(1*R*, 3*S*, 4*R*)-4-(methoxymethoxy)cyclopentane-1,3-diyl]biscarbamate (120 g, 304 mmol) in methanol (420 mL), 4 mol/L hydrogen chloride/1,4‑dioxane solution (420 mL, 1670 mmol) was added, and the mixture was allowed to stand at room temperature for 30 min. The reaction solution was concentrated under reduced pressure to give benzyl [(1*R*, 3*S*, 4*R*)-3-amino-4-hydroxycyclopentyl]carbamate hydrochloride (87.2 g, 100%) as a pale brown solid. ^1^H-NMR (DMSO-D_6_) δ: 1.51-1.70 (2H, m), 2.15-2.29 (2H, m), 3.20-3.35 (1H, m), 3.72-4.14 (3H, m), 5.01 (2H, s), 7.27-7.49 (5H, m), 8.00 (3H, s). MS (m/z): 251 (M+H)^+^.

**Preparation of benzyl {(1*R*, 3*R*, 4*S*)-3-hydroxy-4-[(2-nitrobenzene-1-sulfonyl)amino]cyclopentyl}carbamate**

To a solution of benzyl [(1*R*, 3*S*, 4*R*)-3-amino-4-hydroxycyclopentyl]carbamate hydrochloride (87.2 g, 304 mmol) in dichloromethane (1000 mL), 2-nitrobenzenesulfonyl chloride (74.5 g, 319 mmol) and *N*,*N*-diisopropylethylamine (159 mL, 912 mmol) were added, and the mixture was stirred at 0°C for 1 h. Water was added to the reaction solution, and the mixture was extracted with dichloromethane. The organic layer was washed with saturated brine, dried over anhydrous sodium sulfate, and concentrated under reduced pressure. Ethyl acetate was added to the residue, and the solid was collected by filtration to give benzyl {(1*R*, 3*R*, 4*S*)-3-hydroxy-4-[(2-nitrobenzene-1-sulfonyl)amino]cyclopentyl}carbamate as a colorless solid. The filtrate was concentrated, ethyl acetate was added to the residue, and the solid was collected by filtration to give a colorless solid as the second crop. The filtrate was concentrated, and the residue was purified by amino-silica gel column chromatography (n-hexane/ethyl acetate) to give a pale yellow solid as the third crop. In total, benzyl {(1*R*, 3*R*, 4*S*)-3-hydroxy-4-[(2-nitrobenzene-1-sulfonyl)amino]cyclopentyl}carbamate (99.1 g) was obtained at a 75% yield.^1^H-NMR (CDCl_3_) δ: 1.38-1.88 (2H, m), 2.08-2.34 (2H, m), 3.42 (1H, br s), 3.59-3.73 (1H, m), 3.86-4.09 (2H, m), 5.05 (2H, s), 5.29 (1H, d, J = 6.1 Hz), 6.00 (1H, br s), 7.29-7.51 (5H, m), 7.72-7.76 (2H, m), 7.86-7.89 (1H, m), 8.13-8.16 (1H, m).

**Preparation of benzyl {(1*R*, 3*R*, 4*S*)-3-hydroxy-4-[methyl(2-nitrobenzene-1-sulfonyl)amino]cyclopentyl}carbamate**

To a stirred solution of benzyl {(1*R*, 3*R*, 4*S*)-3-hydroxy-4-[(2-nitrobenzene-1-sulfonyl)amino]cyclopentyl}carbamate (87.2 g, 220 mmol) in *N*,*N*-dimethylformamide (630 mL), cesium carbonate (74.2 g, 220 mmol) and methyl iodide (21.3 mL, 331 mmol) were added at 0°C. After stirring at room temperature for 2 h, water was added to the reaction mixture and the mixture was extracted twice with ethyl acetate. The combined organic layers were washed thrice with washed and saturated brine, dried over anhydrous sodium sulfate, and concentrated under reduced pressure. Ethyl acetate/n-hexane was added to the residue, and the solid was collected by filtration to give benzyl {(1*R*, 3*R*, 4*S*)-3-hydroxy-4-[methyl(2-nitrobenzene-1-sulfonyl)amino]cyclopentyl}carbamate (80.9 g, 82%) as a pale yellow solid. ^1^H-NMR (CDCl_3_) δ: 1.60-1.69 (1H, m), 1.96-2.09 (1H, m), 2.16-2.33 (2H, m), 2.98-3.11 (4H, m), 3.89-4.06 (2H, m), 4.22-4.30 (1H, m), 5.08 (2H, s), 5.26 (1H, d, J = 7.3 Hz), 7.29‑7.41 (5H, m), 7.61-7.76 (3H, m), 8.02-8.05 (1H, m). MS (m/z): 450 (M+H)^+^.

**Preparation of benzyl [(1*R*, 3*R*, 4*S*)-3-hydroxy-4-(methylamino)cyclopentyl]carbamate**

To a stirred solution of benzyl {(1*R*, 3*R*, 4*S*)-3-hydroxy-4-[methyl(2-nitrobenzene-1-sulfonyl)amino]cyclopentyl}carbamate (40.4 g, 89.9 mmol) in tetrahydrofuran (140 mL) and methanol (140 mL), cesium carbonate (52.7 g, 162 mmol) and 4-isopropylbenzenethiol (16.8 mL, 108 mmol) were added. After stirring at room temperature for 3 h, amino-silica gel was added to the reaction solution, and the mixture was concentrated under reduced pressure. The residue was purified by amino-silica gel column chromatography (n-hexane/ethyl acetate, followed by ethyl acetate/methanol) to give an oil. Ethyl acetate/n-hexane was added, and the solid was collected by filtration to give benzyl [(1*R*, 3*R*, 4*S*)-3-hydroxy-4-(methylamino) cyclopentyl]carbamate (22.0 g, 93%) as a yellow solid. ^1^H-NMR (CDCl_3_) δ: 1.28-1.39 (1H, m), 1.78-1.89 (1H, m), 1.91-2.04 (1H, m), 2.33-2.48 (4H, m), 2.78-2.88 (1H, m), 3.95-4.03 (1H, m), 4.11-4.24 (1H, m), 5.07 (2H, s), 5.54 (1H, d, J = 9.2 Hz), 7.28‑7.40 (5H, m). MS (m/z): 265 (M+H)^+^.

**Preparation of benzyl [(1*R*, 3*R*, 4*S*)-3-hydroxy-4-{methyl[6-(2,2,2-trifluoroethyl)thieno[2,3-*d*]pyrimidin-4-yl]amino}cyclopentyl]carbamate**

To a solution of benzyl [(1*R*, 3*R*, 4*S*)-3-hydroxy-4-(methylamino)cyclopentyl]carbamate (43.3 g, 0.164 mol), 4-chloro-6-(2,2,2-trifluoroethyl)thieno[2,3-*d*]pyrimidine (43.5 g, 0.172 mol) in 2-propanol (820 mL), *N*,*N*-diisopropylethylamine (57.1 mL, 0.328 mol) was added, and the mixture was stirred at 90°C for 5 h. The reaction solution was cooled to room temperature; then concentrated under reduced pressure and ethyl acetate, dichloromethane, methanol, and water were added thereto; and the mixture was subjected to liquid separation operation. The organic layer was dried over anhydrous sodium sulfate, filtered, and concentrated under reduced pressure. The obtained solid was suspended in ethyl acetate/n‑hexane, collected by filtration, and dried under reduced pressure to give benzyl [(1*R*, 3*R*, 4*S*)-3-hydroxy-4-{methyl[6-(2,2,2-trifluoroethyl)thieno[2,3-*d*]pyrimidin-4-yl]amino}cyclopentyl]carbamate as a pale yellow solid. The filtrate was concentrated under reduced pressure. The residue was subjected to silica gel column chromatography (ethyl acetate/n-hexane), and the obtained solid was suspended in ethyl acetate. The insoluble substance was collected by filtration and dried to give a pale yellow solid as second crop. In total, benzyl [(1*R*, 3*R*, 4*S*)-3-hydroxy-4-{methyl[6-(2,2,2-trifluoroethyl)thieno[2,3-*d*]pyrimidin-4-yl]amino}cyclopentyl]carbamate (94.4 g) was obtained with quantitative yield. ^1^H-NMR (CDCl_3_) δ: 1.72-1.84 (1H, m), 2.22-2.44 (3H, m), 3.49 (3H, s), 3.62 (2H, q, J = 10.2 Hz), 3.90-3.98 (1H, m), 4.08-4.19 (1H, m), 4.54-4.62 (1H, m), 4.65-4.73 (1H, m), 5.12 (2H, s), 5.46 (1H, d, J = 7.9 Hz), 7.31-7.42 (6H, m), 8.35 (1H, s). MS (m/z): 481 (M+H)^+^.

**Preparation of (1*R*, 2*S*, 4*R*)-4-amino-2-{methyl[6-(2,2,2-trifluoroethyl)thieno[2,3-*d*]pyrimidin-4-yl]amino}cyclopentan-1-ol**

To a solution of benzyl [(1*R*, 3*R*, 4*S*)-3-hydroxy-4-{methyl[6-(2,2,2-trifluoroethyl)thieno[2,3-*d*]pyrimidin-4-yl]amino}cyclopentyl]carbamate (8.98 g, 18.7 mmol) in acetonitrile (90 mL), iodotrimethylsilane (8.09 mL, 56.1 mmol) was added at 0°C, and the mixture was stirred at 0°C for 30 min. Hydrochloric acid (1 N, 3.60 mL, 3.60 mmol) and water were added to the reaction solution, and the mixture was washed with ethyl acetate. The organic layer was extracted with 1 N hydrochloric acid, 2 N aqueous sodium hydroxide solution was added to the combined aqueous layers, and the mixture was extracted 9 times with dichloromethane/methanol. The combined organic layers were dried over anhydrous sodium sulfate and concentrated under reduced pressure to give (1*R*, 2*S*, 4*R*)-4-amino-2-{methyl[6-(2,2,2-trifluoroethyl)thieno[2,3-*d*]pyrimidin-4-yl]amino}cyclopentan-1-ol (6.53 g, 99.9%) as a yellow solid. ^1^H-NMR (CDCl_3_) δ: 1.65-1.73 (1H, m), 1.80-1.91 (1H, m), 2.03-2.12 (1H, m), 2.25-2.36 (1H, m), 3.56 (3H, s), 3.58-3.73 (3H, m), 4.46-4.52 (1H, m), 5.06-5.16 (1H, m), 7.41 (1H, s), 8.39 (1H, s). MS (m/z): 347 (M+H)^+^.

**Preparation of 6-chloro-3,4-dimethoxypyridazine**

3,4,6-Trichloropyridazine (2.57 g, 14.0 mmol) was dissolved in methanol (50 mL), and sodium methoxide (1.56 g, 28.9 mmol) was added thereto. The mixture was stirred at 0°C for 10 min, allowed to warm to room temperature, and stirred for 20 h. The reaction solution was concentrated under reduced pressure, ethyl acetate and saturated aqueous ammonium chloride solution were added thereto, and the mixture was subjected to liquid separation operation. The organic layer was washed with saturated brine, dried over anhydrous sodium sulfate, filtered, and concentrated under reduced pressure. The residue was subjected to silica gel column chromatography (dichloromethane/ethyl acetate), and the obtained solid was suspended in n-hexane/ethyl acetate. The insoluble substance was collected by filtration and dried to give 6-chloro-3,4-dimethoxypyridazine (1.10 g, 45%) as a colorless solid. ^1^H-NMR (CDCl_3_) δ: 3.95 (3H, s), 4.16 (3H, s), 6.77 (1H, s). MS: m/z 175, 177 (M+H)^+^.

**Preparation of 4-(5,6-dimethoxypyridazin-3-yl)benzaldehyde**

To a solution of 6-chloro-3,4-dimethoxypyridazine (1.10 g, 6.30 mmol), 4‑(4,4,5,5‑tetramethyl-1,3,2-dioxaborolan-2-yl)benzaldehyde (2.17 g, 9.35 mmol) in 1,2‑dimethoxyethane (30 mL) and water (10 mL), tetrakis(triphenylphosphine)palladium (0) (0.720 g, 0.623 mmol) and sodium carbonate (1.70 g, 16.0 mmol) were added, and the mixture was stirred at 100°C for 5 h under nitrogen atmosphere. The reaction solution was allowed to cool to room temperature, saturated brine was added thereto, and the mixture was extracted with ethyl acetate. The organic layer was dried over anhydrous sodium sulfate, filtered, and concentrated under reduced pressure. The residue was subjected to silica gel column chromatography (n-hexane/ethyl acetate, followed by ethyl acetate/methanol), and the obtained solid was suspended in n-hexane/ethyl acetate. The resulting solid was collected by filtration and dried to give 4-(5,6-dimethoxypyridazin-3-yl)benzaldehyde (1.21 g, 79%) as a colorless solid. ^1^H-NMR (CDCl_3_) δ: 4.05 (3H, s), 4.26 (3H, s), 7.20 (1H, s), 7.99-8.04 (2H, m), 8.15-8.20 (2H, m), 10.10 (1H, s).

**Preparation of (1*R*, 2*S*, 4*R*)-4-({[4-(5,6-dimethoxypyridazin-3-yl)phenyl]methyl}amino)-2-{methyl[6-(2,2,2-trifluoroethyl)thieno[2,3-*d*]pyrimidin-4-yl]amino}cyclopentan-1-ol (DS-1594a)**

To a stirred solution of (1*R*, 2*S*, 4*R*)-4-amino-2-{methyl[6-(2,2,2-trifluoroethyl)thieno[2,3-*d*]pyrimidin-4-yl]amino}cyclopentan-1-ol (0.102 g, 0.294 mmol) in dichloromethane (3.2 mL), 4-(5,6-dimethoxypyridazin-3-yl)benzaldehyde (0.0844 g, 0.346 mmol), sodium triacetoxyborohydride (0.215 g, 0.101 mmol), and acetic acid (0.0500 mL, 0.874 mmol) were added, and the mixture was stirred at room temperature for 17 h. Water/saturated aqueous sodium bicarbonate solution (2/1) was added to the reaction solution, and the mixture was extracted with dichloromethane/methanol (9/1). The organic layer was dried over anhydrous sodium sulfate, filtered, and concentrated under reduced pressure, and the residue was purified by silica gel column chromatography (dichloromethane/methanol) to give (1*R*, 2*S*, 4*R*)-4-({[4-(5,6-dimethoxypyridazin-3-yl)phenyl]methyl}amino)-2-{methyl[6-(2,2,2-trifluoroethyl)thieno[2,3-*d*]pyrimidin-4-yl]amino}cyclopentan-1-ol (0.115 g, 59%) as a colorless caramel. ^1^H-NMR (CDCl_3_) δ: 1.80-1.86 (1H, m), 1.94-2.03 (1H, m), 2.10 (1H, dt, J = 13.9, 5.7 Hz), 2.28-2.38 (1H, m), 3.38-3.45 (1H, m), 3.53 (3H, s), 3.61 (2H, q, J = 10.2 Hz), 3.89 (1H, d, J = 13.2 Hz), 3.93 (1H, d, J = 13.2 Hz), 4.00 (3H, s), 4.23 (3H, s), 4.46-4.51 (1H, m), 5.04 (1H, td, J = 9.7, 4.7 Hz), 7.11 (1H, s), 7.38 (1H, s), 7.44 (2H, d, J = 8.0 Hz), 7.96 (2H, d, J = 8.0 Hz), 8.40 (1H, s). MS (m/z):575 (M+H)^+^.

**Preparation of (1*R*, 2*S*, 4*R*)-4-({[4-(5,6-dimethoxypyridazin-3-yl)phenyl]methyl}amino)-2-{methyl[6-(2,2,2-trifluoroethyl)thieno[2,3-*d*]pyrimidin-4-yl]amino}cyclopentan-1-ol hydrochloride (DS-1594a·HCl)**

(1*R*, 2*S*, 4*R*)-4-({[4-(5,6-dimethoxypyridazin-3-yl)phenyl]methyl}amino)-2-{methyl[6-(2,2,2-trifluoroethyl)thieno[2,3-*d*]pyrimidin-4-yl]amino}cyclopentan-1-ol (0.112 g, 0.170 mmol) was dissolved in ethanol (3.60 mL), and 1 mol/mL hydrochloric acid ethanol solution (0.173 mL, 0.173 mmol) was added thereto. The mixture was concentrated under reduced pressure. The obtained solid was suspended in diethyl ether, collected by filtration, and dried under reduced pressure to give (1*R*, 2*S*, 4*R*)-4-({[4-(5,6-dimethoxypyridazin-3-yl)phenyl]methyl}amino)-2-{methyl[6-(2,2,2-trifluoroethyl)thieno[2,3-*d*]pyrimidin-4-yl]amino}cyclopentan-1-ol hydrochloride (0.104 g, 98%) as a pale yellow solid. 1H-NMR (DMSO-D6) δ: 1.80-1.89 (1H, m), 2.26-2.36 (1H, m), 2.43-2.54 (2H, m), 3.44 (3H, s), 3.55‑3.66 (1H, m), 4.01 (3H, s), 4.03-4.16 (5H, m), 4.22-4.40 (3H, m), 4.90-5.00 (1H, m), 5.20 (1H, d, J = 4.3 Hz), 7.71-7.79 (3H, m), 8.19 (1H, d, J = 8.0 Hz), 8.36 (1H, s), 9.47 (1H, br s), 9.53 (1H, br s). MS (m/z): 575 (M+H)^+^. [α]_D_^20^ -23.5 (c = 1.00, MeOH).

**Preparation of (1*R*, 2*S*, 4*R*)-4-({[4-(5,6-dimethoxypyridazin-3-yl)phenyl]methyl}amino)-2-{methyl[6-(2,2,2-trifluoroethyl)thieno[2,3-*d*]pyrimidin-4-yl]amino}cyclopentan-1-ol succinate (DS-1594a·succinate, DS-1594b)**

(1*R*, 2*S*, 4*R*)-4-({[4-(5,6-dimethoxypyridazin-3-yl)phenyl]methyl}amino)-2-{methyl[6-(2,2,2-trifluoroethyl)thieno[2,3-*d*]pyrimidin-4-yl]amino}cyclopentan-1-ol (25.8 g, 44.9 mmol) was suspended in 2-propanol (103 mL), and water (12.9 mL) was added thereto. Succinic acid (5.56 g, 47.1 mmol) was added to the mixture followed by water (12.9 mL), and the mixture was stirred at room temperature for 4 h. The insoluble substance was collected by filtration while washing with 2-propanol (150 mL) and dried to give (1*R*, 2*S*, 4*R*)-4-({[4-(5,6-dimethoxypyridazin-3-yl)phenyl]methyl}amino)-2-{methyl[6-(2,2,2-trifluoroethyl)thieno[2,3-*d*]pyrimidin-4-yl]amino}cyclopentan-1-ol succinate (30.1 g, 97%) as colorless crystals. ^1^H-NMR (DMSO-D_6_) δ: 1.50-1.58 (1H, m), 2.04-2.19 (2H, m), 2.27‑2.35 (1H, m), 2.37 (4H, s), 3.14-3.24 (1H, m), 3.42 (3H, s), 3.88-3.94 (2H, m), 3.99 (3H, s), 4.05-4.11 (5H, m), 4.28-4.33 (1H, m), 4.80-4.90 (1H, m), 7.54 (2H, d, J = 8.5 Hz), 7.61 (1H, s), 7.72 (1H, s), 8.08 (2H, d, J = 8.5 Hz), 8.33 (1H, s). MS (m/z): 575 (M+H)^+^. [α]_D_^20^ -28.3 (c = 1.00, DMSO).

**Expression and purification of human menin.**

For crystallographic study of menin and DS‑1594a·succinate and for menin-MLL1 interaction assays, the expression and purification of human menin were performed as described previously^1^ with some modifications. Briefly, full-length menin (isoform 1) with a His_6_-small ubiquitin-like modifier (SUMO) tag at the N-terminus and a FLAG tag at the C‑terminus or menin with deletion of residues 460–519 (isoform 2) with a His_6_-SUMO tag at the N-terminus was expressed in *E. coli*. The proteins were purified by nickel affinity chromatography, and the N-terminal tags were cleaved with sentrin-specific protease 2 (SENP2) before a second nickel affinity chromatography step, anion-exchange chromatography (for isoform 2 only), and further size-exclusion chromatography with a buffer containing 25 mM Tris (pH 8.0), 150 mM sodium chloride (NaCl), and 5 mM DTT.

**Crystal preparation and structure determination.**

Purified human menin was mixed with precipitant solution containing 0.1 M cacodylate·HCl (pH 6.4), 1.5 M sodium acetate (NaOAc), and 20 mM barium chloride (BaCl_2_) and vapor-equilibrated with the same solution at 10°C. After 2 months, crystals of human apo menin were grown to the appropriate size for the X-ray experiment. DS-1594a·succinate was introduced to apo menin crystals by soaking in a solution containing 0.1 M Bis-Tris propane·HCl (pH 7), 1.28 M NaOAc, 5 mM BaCl_2_, 20% (v/v) glycerol, and 0.5 mM DS‑1594a·succinate at 10°C overnight. Diffraction data were collected on BL-1A at Tsukuba Photon Factory and processed with *XDS*^2^ and *AIMLESS*.^3^ Molecular replacement, phase refinement, and model building were carried out using *Phaser*,^4^ *REFMAC5*,^5^ and *COOT*,^6^ respectively. The statistics of data processing and phase refinement are summarized in Supplemental Table 1. Figures describing the crystal structures were drawn with *PyMOL*.^7^

**Additional file 1: Table S1. Statistics for data collection and phase refinement**

| **Data collection** | |
| --- | --- |
| Space group | *P*2_1_ |
| Cell dimensions |  |
| *a, b, c* (Å) | 141.58, 70.15, 144.43 |
| *α, β, γ* (°) | 90, 91.5, 90 |
| Resolution (Å) | 49.8-2.6 (2.65-2.60) |
| *R*merge(*I*) (%) | 8.3 (127.3) |
| *I*/σ*I* | 3.6 (0.2) |
| No. of reflections | 289542 (13705) |
| Unique reflections | 87588 (4371) |
| Completeness (%) | 99.8 (98.8) |
| Redundancy | 3.3 (3.1) |
| **Refinement** | |
| Resolution (Å) | 25-2.6 (2.69-2.60) |
| No. of reflections | 83083 |
| *R*work/*R*free (%) | 21.2/26.4 (38.8/38.6) |
| No. of atoms |  |
| Protein | 14717 |
| Ligand/ion | 180 |
| Water | 142 |
| Average B-factor |  |
| Protein | 66.6 |
| Ligand/ion | 48.9 |
| Water | 47.7 |
| Root mean square deviations |  |
| Bond lengths (Å) | 0.004 |
| Bond angles (°) | 1.144 |

The values in parentheses correspond to the highest-resolution shells. To calculate Rfree, 5% of reflections were excluded from the refinement. Rmerge was defined by the equation Rmerge = Σhkl Σj|Ij(hkl) − <I(hkl)>|/Σhkl Σj Ij(hkl).

**Menin-MLL1 interaction assay (AlphaLISA).**

Biotinylated MLL1 peptide 1-2 (biotin-MLL-peptide 1-2: Biotin-MAHSC RWRFP ARPGT TGGGG GGGRR GLGGA PRQRV PALLL PPGPP V-NH2) was purchased from SCRUM, Inc. Full-length menin (isoform 1) with a C‑terminal FLAG tag (menin-FL) was used for this assay. The inhibitory effects of DS‑1594a·succinate on the menin-MLL1 interaction were determined by AlphaLISA assay. The assay was performed using a 384-well plate (AlphaPlate-384 Shallow well, PerkinElmer Inc.), to which menin-FL, biotin-MLL1-peptide 1-2, and 4% (v/v) DMSO were added as controls. The background wells contained only 4% (v/v) DMSO. Five microliters of menin‑MLL1 solution was added to each well except for the background wells. DS‑1594a·succinate was added to achieve final concentrations of 1000, 170, 28, 4.6, 0.77, 0.13, 0.021, and 0.0036 nM in all wells, excluding the background and control wells. The plate was covered, mixed on a plate shaker for 1 min, centrifuged for 1 min at 1000 rpm, and incubated for 30 min at room temperature. Subsequently, the detection reagent (50 mM Tris-hydrochloride [HCl, pH 7.5], 50 mM NaCl, 0.01% (v/v) BSA, 0.01% (w/v) Triton X-100, 3 mM Tris(2-chloroethyl) phosphate (TCEP), 20 μg/mL anti-FLAG AlphaLISA acceptor beads, and 20 μg/mL streptavidin-coated AlphaScreen donor beads) was added to each well under dark conditions, and the aluminum-sealed plate was incubated for 60 min at room temperature after brief mixing and centrifugation for 1 min at 1000 rpm. Emission was measured at 570 nm with an EnVision reader (PerkinElmer Inc., Model: 2014-0020, Serial No. 1040374) using the AlphaScreen program. The inhibition curve for DS-1594a·succinate was estimated according to the sigmoid Emax model, and the sigmoid curve was drawn using GraphPad Prism (version 4.03). The crosstalk correction value was automatically calculated and was used for estimation of the half-maximal inhibitory concentration (IC_50_).

**Coimmunoprecipitation experiments.**

To confirm the menin-MLL1 interaction, coimmunoprecipitation of menin and MLL1 was performed. HEK293T cells (Thermo Fisher Scientific, Inc.) were transfected with HA-human MLL1N_1-1395_ plasmid using Lipofectamine 2000 (Thermo Fisher Scientific, Inc.). One day after transfection, the cells were treated with DMSO or DS-1594a·HCl (0.1, 0.3, 1.0 μM in 0.1% [v/v] final DMSO concentration) for 1 day. Whole cells were resuspended in lysis buffer (20 mM Tris-HCl [pH 8.0], 0.1% (w/v) NP-40, 300 mM potassium chloride [KCl], 1 mM EDTA, 10% [v/v] glycerol) supplemented with phosphatase inhibitor and protease inhibitor, immunoprecipitated with anti–HA-tag antibody (Santa Cruz Biotechnology, Inc.), and analyzed by SDS‒PAGE and Western blotting.

**Cell lines and patient-derived primary AML cells.**

HEK293T cells were cultured in DMEM (Thermo Fisher Scientific, Inc.) supplemented with 10% (v/v) FBS (GE Healthcare Life Science). In all patient-derived cell lines, *MLL1* rearrangement was identified at diagnosis of AML. AML#8531 cells were cultured in hematopoietic stem cell expansion medium (Stemline II HSCEM, Sigma‒Aldrich) supplemented with IL-3 (Miltenyi Biotec), IL-6 (Miltenyi Biotec), stem cell factor (SCF; Miltenyi Biotec), thrombopoietin (TPO; Miltenyi Biotec), and FMS-like tyrosine kinase 3 ligand (FLT3L; Miltenyi Biotec) at 20 ng/mL each. AML676 and NCCHD010 cells were cultured in Stemline II HSCEM supplemented with 20 ng/mL IL-3 (PeproTech), 20 ng/mL IL-6 (PeproTech), 100 ng/mL SCF (PeproTech), 100 ng/mL TPO (PeproTech), and 100 ng/mL FLT3L (PeproTech). All cells were cultured in a carbon dioxide (CO_2_) incubator (5% CO_2_, 37°C, humidity-conditioned).

**Cell viability assay and staining in human leukemic cell lines.**

To assess cell viability in human leukemic cell lines, a CellTiter-Glo 2.0 cell viability assay (G9243, Promega Corporation) was performed following trypan blue-based cell density assessment by mixing diluted cells of each cell line with 0.2% (v/v) DMSO solution with or without DS-1594a·HCl in a 96-well plate. CellTiter-Glo was added on day 0 and day 7, after which the luminescence intensity was measured using a plate reader (EnVision 2104 Multilabel Reader, PerkinElmer Japan Co., Ltd., Serial number: 1040374). The GI_50_ was defined as the concentration of DS‑1594a·HCl at which the cell survival rate was 50%.

**Establishment of human *MLL-AF9*–evoked murine AML-like (murine MA9) cells.**

Murine c‑KIT+ bone marrow cells transduced with MLL-AF9 were cultured and maintained in 2 different media (1. StemPro-34 SFM [Gibco] supplemented with GlutaMAX I [Gibco], penicillin‒streptomycin [Gibco], 10 ng/mL human oncostatin M [Wako], 10 ng/mL mouse IL-3 [PeproTech], and 50 ng/mL mouse SCF [PeproTech]; 2. IMDM supplemented with 20% [v/v] FBS, GlutaMAX I, penicillin‒streptomycin, 55 μM 2-mercaptoethanol [Gibco], nonessential amino acid solution [Gibco], 30 ng/mL human IL-6 [PeproTech], 20 ng/mL mouse IL-3, and 50 ng/mL mouse SCF) over 3–6 months.

**May-Grünwald-Giemsa (MGG) staining in murine MA9 cells.**

Microscopic examination of cell differentiation in murine MA9 cells was carried out using MGG staining. Cells were harvested 7 days after the addition of DMSO, DS-1594a·HCl (final concentration of 10, 20, or 40 nM), or Ara-C (final concentration of 50 or 100 nM) to the medium and stained using Giemsa solution for microscopic observation. Murine MA9 cells were harvested 7 days after the addition of DMSO, DS-1594a·HCl (final concentration of 10, 20, or 40 nM), or Ara-C (final concentration of 50 or 100 nM) to the medium. For staining, cells in 100 μL of medium (2 × 10^6^ cells/mL) were plated on glass slides by centrifugation (300 rpm, 2 min at room temperature) with a cytospin, and the cells were dried at room temperature for 1 h. The slides were serially immersed in May-Grünwald stain (Muto Chemical Co., Ltd.) for 2 min, May-Grünwald diluent (May-Grünwald stain diluted 1/10 with 1/150 M phosphate buffer [pH 6.4]) for 4 min, and Giemsa solution (Muto Chemical Co., Ltd.; diluted 1/33 using 1/150 M phosphate buffer [pH 6.4]) for 12 min and then washed off with tap water for 20 s, dried with cold air, and observed under a microscope.

**Cellular growth assay in murine MA9 cells with washout of compound.**

To test the maintenance of drug efficacy in vitro, murine MA9 cells (1.5 or 3 × 10^4^ cells/mL) were seeded at 2 mL/well in 6-well plates and cultured at 37°C and 5% CO_2_ for 7 days with 2 μL of DMSO, DS-1594a·HCl (final concentration of 10, 20, or 40 nM), or Ara-C (final concentration of 50, 100, or 200 nM). The cells were harvested, washed with PBS, and cultured for another 7 days without the test compounds. Cell viability was measured on days 7, 10, and 14 with a CellTiter-Glo 2.0 cell viability assay, and a colony‑forming assay was performed on day 13 using MethoCult GF M3434 as described before. All experiments were performed in triplicate.

**Colony-forming unit assay in murine MA9 cells using MethoCult and serial plating.**

To test the colony-forming and self-replicating ability of murine MA9 cells upon DS-1594a·HCl and Ara-C treatment, murine MA9 cells were counted using a TC10 fully automated cell counter (Bio-Rad, cat. no. 145-0001J1), and diluted cell fluids were prepared at 1 × 10^4^ cells/mL using 2% (v/v) FBS‑containing IMDM. The cells were plated on methylcellulose media supplemented with human cytokines (MethoCult^TM^ GF M3434, STEMCELL Technologies) along with a mixed solution of DMSO, DS-1594a·HCl (final concentration of 10, 20, or 40 nM), or Ara-C (final concentration of 50 or 100 nM) and incubated at 37°C under 5% CO_2_ and 95% humidity for 4 days, after which colonies that formed were counted under a microscope. The colonies were disrupted, and cells were replated in serial dilutions for repeat MethoCult assays every 4–5 days for 4 cycles. Murine MA9 cells were counted using a TC10 fully automated cell counter (Bio-Rad, cat. no. 145-0001J1), and diluted cell fluids were prepared at 1 × 10^4^ cells/mL using a 2% (v/v) FBS-containing IMDM. Next, 300 μL of diluted cell fluid and 3 μL of a mixed solution of DMSO, DS-1594a·HCl (final concentration of 10, 20, or 40 nM), or Ara-C (final concentration of 50 or 100 nM) were added to MethoCul GF M3434 (STEMCELL Technologies) that had previously been dispensed in 3 mL portions in 14 mL round tubes and mixed vigorously using a vortex. After the tubes were allowed to stand for a minimum of 5 min, 1.2 mL was added to each of two 35 mm culture dishes (STEMCELL Technologies) using a 16 gauge blunt-end needle (STEMCELL Technologies) and 3 mL syringes (Terumo), and the cells were cultured in a 5% CO_2_ incubator at 37°C and 95% or more humidity. The colonies that formed from more than 30 populations of cells under microscopic observation after 4 days were counted (MC1). After colony counting, 2 mL/dish PBS was added for serial replating, and after the colonies were removed with a scraper, the cells were resuspended by pipetting and then transferred to 15 mL tubes. One milliliter PBS/dish was added again, pipetted, and transferred to the same 15 mL tube; the tube was placed on ice. After centrifugation (1000 rpm, 5 min, 4°C), the supernatant was removed, 1 mL of PBS was added, and the tube was centrifuged again (1000 rpm, 5 min, 4°C). The supernatants were removed and then suspended in 125, 250, 500, or 1000 μL of 2% (v/v) FBS-containing IMDM, and the cells were counted using a TC10 fully automated cell counter (Bio-Rad, cat. no. 145-0001J1). Diluted cells were prepared and used again for MethoCult assays as in MC1. The above was repeated 4 more times, and the numbers of colonies were counted 4–5 days after seeding (MC2, MC3, MC4, MC5).

**FCM analysis of human *MLL-AF9*–evoked murine AML-like (murine MA9) cells:** Murine MA9 cells were harvested 7 days after the addition of DMSO, DS-1594a·succinate (final concentration of 10, 20, or 40 nM), or Ara-C (final concentration of 50 or 100 nM) to the medium and counted using a TC20 fully automated cell counter (Bio-Rad, cat. no. 145-0109J1). Next, 1–5 × 10^6^ cells were transferred into 1.5 mL tubes and centrifuged (3500 rpm, 3 min, 4°C). The cell pellet was resuspended in 1 mL of SM buffer (PBS with 5% [v/v] FBS), to which 5 μL of mouse BD Fc Block resuspended in 50 μL of SM buffer was added, and the mixture was incubated for 10 min at room temperature. Five antibodies (shown in the table below) were added in 0.5 μL increments and incubated on ice for 30 min. Only for samples to which Annexin V-phycoerythrin (PE) was added, 100 μL of SM buffer was added, the sample was centrifuged (3500 rpm, 3 min, 4°C), the supernatant was removed and suspended in 100 μL of Annexin V-PE containing 1 x Annexin V binding buffer (40 μL of Annexin V-PE added to 800 μL of 1 x Annexin V binding buffer), the mixture was incubated at room temperature for 15 min, 400 μL of 1 x Annexin V binding buffer was added, the sample was centrifuged (3500 rpm, 3 min, 4°C), and the supernatant was removed. Finally, 0.5 μL of 0.5 mg/mL DAPI (Donin Chemical Laboratory, cat. no. D523)-containing SM buffer was added to Mix 1 and Mix 2 samples in increments of 400 μL and 500 μL, respectively, and the samples were analyzed using a flow cytometer (Novocyte 3000, ACEA Biosciences, Inc.) through a tube with a 35-μm cell strainer (Corning, cat. no. 352235).

**Colony-forming unit assay in human cord blood-derived CD34+ cells using MethoCult H4535.**

To test the colony-forming and self-replicating ability of human cord blood-derived CD34+ cells (ALLCELLS、Cat. No. CB005F) upon DS-1594a·HCl and Ara-C treatment, the cells were counted using a TC20 fully automated cell counter (Bio-Rad), and diluted cell fluids were prepared at 2 × 10^4^ cells/mL using 2% (v/v) FBS‑containing IMDM (Thermo Fisher Scientific Inc.). The cells were plated on methylcellulose media (MethoCult H4535, STEMCELL Technologies) along with a mixed solution of DMSO, DS-1594a·HCl (final concentration of 1,000, 3,000, or 10,000 nM), or Ara-C (final concentration of 40 or 200 nM) and incubated at 37°C under 5% CO_2_ and 95% humidity for 12 days, after which colonies that formed were counted under a microscope.

**Additional file 1: Table S2.** **Antibodies used for FCM in murine MA9 cells**

| **Antibody** | **Manufacturer** | **Cat. no.** |
| --- | --- | --- |
| Mouse BD Fc Block | BD Biosciences | 553142 |
| FITC anti-mouse Ly-6G antibody | BioLegend | 108405 |
| APC anti-mouse/human CD11b antibody | BioLegend | 101212 |
| PE anti-mouse Ly-6C antibody | BioLegend | 128008 |
| FITC anti-mouse Ly-6A/E antibody | BioLegend | 108106 |
| APC anti-mouse/human CD117 (c-KIT) antibody | BioLegend | 105812 |
| Annexin V-PE | BD Biosciences | 556421 |

APC, allophycocyanin; FCM, flow cytometry; PE, phycoerythrin.

**Additional file 1: Table S3. Sample types used for FCM in murine MA9 cells**

| **Sample type** | **Antibody 1** | **Antibody 2** | **Antibody 3** | **Antibody 4** |
| --- | --- | --- | --- | --- |
| Compensation #1 | - | - | - | - |
|  | Ly-6G-FITC | - | - | - |
|  | - | Ly6C-PE | - | - |
|  | - | - | CD11b-APC | - |
|  | - | - | - | DAPI |
| Mix 1 | Ly-6G-FITC | Ly6C-PE | CD11b-APC | DAPI |
| Compensation #2 | - | - | - | - |
|  | Ly-6A/E-FITC | - | - | - |
|  | - | Annexin V-PE | - | - |
|  | - | - | CD117-APC | - |
|  | - | - | - | DAPI |
| Mix 2 | Ly-6A/E-FITC | Annexin V-PE | CD117-APC | DAPI |

APC, allophycocyanin; FCM, flow cytometry; PE, phycoerythrin.

**cDNA library preparation for RNA-seq and whole-transcriptome analysis.**

Total RNA was examined for quality and quantity with a 4200 TapeStation (Agilent Technologies, Inc.) and Qubit instrument (Thermo Fisher Scientific, Inc.), respectively. The cDNA library for RNA-seq was prepared with a QIAseq FastSelect kit (rRNA depletion, QIAGEN, Inc.), an NEBNext Ultra II Directional RNA Library Prep Kit for Illumina (New England Biolabs, Inc.), and NEBNext Multiplex Oligos for Illumina (New England Biolabs, Inc.). The prepared libraries were pooled and sequenced at 1.5 nM on a NovaSeq 6000 (Illumina, Inc.) with 75 bp paired-end reads (2 × 75 bp). The reads obtained were mapped to the reference human genome using STAR software (<https://github.com/alexdobin/STAR>/, version 2.5.2a) implemented in the Genomon pipeline (<https://github.com/Genomon-Project/>, version 2.6.3) with its default settings. The mapped reads were counted for each gene and normalized with the R package DESeq2 (version 1.32.0). GSEA^8^ was performed using the GSEA online tool (<https://www.gsea-msigdb.org/gsea/index.jsp>, version 4.1.0). The MLL-AF9 target gene set derived from Bernt et al.^9^ as well as “curated gene sets” (C2.all.v7.4) from MSigDB collections^10,11^ were used in GSEA. An mRNA expression signature enriched in LSCs was used to create an LSC17 score^12^ and pLSC6 score^13^; patients with high LSC17 scores had poor outcomes with current treatments, including allogeneic stem cell transplantation. The RNA-seq raw count data were normalized between samples with the edgeR Bioconductor package^14^. The LSC score was calculated as the weighted sum of the expression of 17 genes for LSC17 and 6 genes for pLSC6.

**ChIP.**

Cells were harvested after 3 days of treatment with the test compounds. Cell fixation and chromatin fragmentation were performed using a SimpleChIP^®^ Enzymatic Chromatin IP Kit (Cell Signaling Technology), and ChIP and purification of DNA were performed using a MAGnify^™^ Chromatin Immunoprecipitation System (Applied Biosystems). The antibodies used for ChIP are described in Supplemental Table 4.

**Library preparation for ChIP-seq and analysis.**

Input and ChIP DNA samples with their respective antibodies were quantified with a Qubit instrument (Thermo Fisher Scientific, Inc.). ChIP-seq libraries were prepared using a ThruPlex DNA-Seq Kit (Clontech, Inc.) and a DNA Single Index Kit (for MOLM-13) or a DNA HT Dual Index Kit (Clontech, Inc.; for NCCHD010) according to the manufacturer’s instructions. The quality and quantity of the prepared libraries were evaluated with a Qubit Bioanalyzer (Agilent Technologies, Inc.) or a 4200 TapeStation (Agilent Technologies, Inc.). The prepared libraries were pooled and sequenced on a NextSeq 500 (Illumina, Inc.) for MOLM-13 cells and a NovaSeq 6000 for NCCHD010 cells.

Sequencing reads were obtained in 75 bp single-end mode for the MOLM-13 samples and in 150 bp paired-end mode for the NCCHD010 samples and mapped to the reference human genome (GRCh37) using Bowtie2 (version 2.3.4.3) with the default parameters. The “count” function of IGVtools in Integrated Genomics Viewer (IGV, version 2.11.1) was used to generate coverage data with a window size of 25 bp. The coverage data were normalized by the total number of reads in each sample and visualized by IGV. For experimental conditions in which cells and antibodies were common, the same scale was used for the vertical axis.

**Additional file 1: Table S4.** **Antibodies used for ChIP**

| **Antibody** | **Manufacturer** | **Cat. no.** |
| --- | --- | --- |
| Anti-menin | Abcam plc | ab31902 |
| Anti-MLL1 | Bethyl Laboratories, Inc. | A300-086A |
| Anti-H3K4me3 | Abcam plc | ab8580 |
| Anti-H3K79me2 | Active Motif | 39143 |

ChIP, chromatin immunoprecipitation; MLL1, mixed-lineage leukemia 1.

**FCM analysis of patient-derived AML samples.**

AML676 and NCHD010 cells were cultured for 7 days with DMSO, DS-1594a·succinate, or Ara-C and then stained with antibodies (Supplemental Table 5) on day 7 using a standard FCM protocol. DMSO or the compound dissolved in DMSO was added to the culture medium at day 0 to achieve a 0.1% final concentration. Human Fc receptors were blocked using Human BD Fc Block. Dead cells were excluded by staining with DAPI (Dojindo). Analysis was performed using a Novocyte 3000 (ACEA Biosciences, Inc.).

AML#8531 (CRB Hospices Civils de Lyon), AML#7789 (ProteoGenex), AML#7915 (CRB Hospices Civils de Lyon), and AML#7919 (CRB Hospices Civils de Lyon) cells were seeded in hematopoietic stem cell expansion medium (Stemline II HSCEM, Sigma‒Aldrich, cat. #S0192) supplemented with IL-3 (Miltenyi Biotec, cat. #130-093-909), IL-6 (Miltenyi Biotec, cat. #130-095-365), SCF (Miltenyi Biotec, cat. #130-093-991), TPO (Miltenyi Biotec, cat. #130-094-011), and FLT3L (Miltenyi Biotec, cat. #130-093-854) at 20 ng/mL each in a 96-well cell culture plate (100 µL/well). Test and reference compounds (dissolved in DMSO) were added at a 1:1000 dilution. The cells were cultured for 7 days with the addition of fresh medium and compounds on day 3 and then stained with antibodies (Supplemental Table 6) using a standard FCM protocol. Human Fc receptors were blocked using Human TruStain FcX Fc Receptor blocking solution (BioLegend, cat. #422302). Dead cells were excluded by staining with a live/dead fixable dye (Thermo Fisher Scientific, cat. #L34975). Analysis was performed using a BD FACS Aria III and FlowJo software (BD Biosciences). The genetic background of the primary samples was described in Supplemental Table 7.

**Additional file 1: Table S5.** **Antibodies used in FCM analysis of human-derived AML676 and NCCHD010 cells**

| **Product name** | **Manufacturer** | **Cat. no.** |
| --- | --- | --- |
| FITC anti-human CD34 | BioLegend | 343604 |
| APC anti-human CD38 | BioLegend | 356606 |
| FITC anti-human CD14 | BioLegend | 301804 |

APC, allophycocyanin; FCM, flow cytometry; PE, phycoerythrin.

**Additional file 1: Table S6.** **Antibodies used in FCM analysis of human-derived AML#8531, AML#7789, AML#7915, and AML#7919 cells**

|  | | **Antibody** | **Isotype control** |
| --- | --- | --- | --- |
| Fluorochrome | Target | Clone (manufacturer; cat. no.) | Name (clone; manufacturer; cat. no.) |
| APC | CD14 | M5E2  (BioLegend; 301808) | mouse IgG2a  (MOPC-173; BioLegend; 400220) |
| BV421 | CD33 | WM53  (BioLegend; 303416) | mouse IgG1  (MOPC-21; BioLegend; 400158) |
| BV650 | CD11b | ICRF44  (BioLegend; 301336) | mouse IgG1  (MOPC-21; BioLegend; 400164) |
| FITC | CD34 | 561  (BioLegend; 343604) | mouse IgG2a  (MPC-173; BioLegend; 400210) |
| PE | CD33  CD34 | WM53  (BioLegend; 303404)  581  (BD Biosciences; 555822) | mouse IgG1  (MOPC-21; BioLegend; 400114) |
| PE/Cy7 | CD38 | LS198-4-3  (Beckman Coulter; B49198) | mouse IgG1  (MOPC-21; BioLegend; 400126) |
| PerCP-Cy5.5 | CD123 | 6H6  (BioLegend; 306016) | mouse IgG1  (MOPC-21; BioLegend; 400150) |

APC, allophycocyanin; FCM, flow cytometry; PE, phycoerythrin.

**Additional file 1: Table S7.** **Information on primary patient samples**

**Animals and survival studies.**

Survival studies in MOLM-13 xenograft model: NSG mice were intravenously injected with 1 × 10^4^ MOLM-13 cells. On day 3, the NSG mice were divided into vehicle and treatment groups of 9 mice per group and were treated for 17 or 19 consecutive days with vehicle (0.5% [w/v] methylcellulose 400 solution, Wako) or DS-1594a·HCl (50, 100, or 200 mg/kg, orally once daily [PO, QD]) for the survival test group and FCM test group. In the FCM test group (n = 3), the mice were sacrificed on day 20 after transplantation, when the vehicle group became moribund. Leukemia was confirmed by FCM analysis of hCD45+ cells using anti-human CD45 (hCD45)-FITC (BioLegend, BD Biosciences), which reflected the population of human leukemic blasts in the bone marrow. In the survival test group (n = 6), the mice were monitored for leukemia progression and were sacrificed upon signs of distress/disease.

Survival studies in the *NPM1c* AML-PDX model (AM7577): NOD/SCID mice (n = 30) were intravenously injected with 2 × 10^6^ AM7577 cells. On day 31, the NOD/SCID mice were split into vehicle and treatment groups of 6 mice per group and were treated with vehicle or DS-1594a·HCl (25, 50, 100, or 200 mg/kg; PO, QD) for 24 or 35 days. The tumor burden in peripheral blood was detected by FCM analysis weekly using anti-human CD45. The mice were terminated on day 35, and their peripheral blood, spleens, and bone marrow cells were collected for FCM analysis.

Survival studies in the *MLL1-r* ALL-PDX model (NCCHD007): Peripheral blood mononuclear cells (1 × 10^6^ cells) from patients with acute lymphocytic leukemia (NCCHD007) with the t(11;19)(q​23.3;p13.3​)​ translocation (*MLL-ENL* fusion gene) were transplanted into the tail veins of NSG mice. Ten days after tail-vein implantation, 3 animals per group for day-38 FCM analysis and 6 animals per group for life-prolongation assessment were randomly divided into 4 groups (the vehicle group and 12.5, 25, and 50 mg/kg DS-1594a·succinate groups; all BID × 28 days). The ILS (%) was calculated by using the following formula = [(median survival time of each group)/median survival time of vehicle control) − 1] × 100. All mice in the day-38 FCM analysis groups were euthanized on day 38; their spleens and hindlimb bones were harvested, and their spleens were weighed. The mice in the life-prolongation efficacy group were euthanized and considered dead at humane endpoints when symptoms of complete paralysis of both hindlimbs, respiratory abnormalities, ≥15% weight loss from the first day of treatment, or moribundity were identified. All treatment-group mice that were alive on day 161 after transplantation were euthanized; their spleens and hindlimb bones were harvested, and their spleens were weighed. FCM analysis was performed for bone marrow cells from the hindlimb bone and peripheral blood cells.

**FCM analysis of bone marrow cells from NCCHD007 mice.**

Hindlimb bones (femurs and tibias) of euthanized mice were harvested, and BM cells within the hindlimb bones were harvested in 1.5 mL tubes containing 5 μL of 0.5 M EDTA by aligning the bone in a 0.5 mL tube with holes at the bottom and by centrifuging (3000 rpm, 1 min, 4°C) the 0.5 mL tube within a 1.5 mL tube. Subsequently, 1 mL of hemolysis buffer (Sigma‒Aldrich, cat. no. R7757) was added, and the mixture was pipetted into a 35-μm cell strainer tube (Corning, cat. no. 352235) and incubated for 1 min at room temperature. After centrifugation (3500 rpm, 3 min, 4°C) of the tube containing the cell suspension, the supernatant was removed, and the pellet was resuspended in 1 mL of SM buffer (PBS containing 5% [v/v] FBS). After the suspension was passed through a tube with a 35-μm cell strainer (Corning, cat. no. 352235), the cell numbers and survival were measured using a TC10 fully automated cell counter (Bio-Rad, cat. no. 145‑0001J1). Approximately 2 × 10^5^ live cells/sample were transferred into 1.5 mL tubes and centrifuged (3500 rpm, 3 min, 4°C). The supernatant was removed, 50 μL of Human BD Fc Block (BD Biosciences, cat. no. 564220) was added (5 μL Human BD Fc Block 5 + 50 μL SM buffer), and the mixture was incubated for 10 min at room temperature. Five microliters of FITC anti-human CD45 antibody (hCD45; BioLegend, cat. no. 368508) was then added, the mixture was incubated for 30 min on ice, and the cells were precipitated by centrifugation (3500 rpm, 3 min, 4°C) after adding 200 μL of SM buffer. After removing the supernatant, 500 μL of SM buffer containing 0.5 μg/mL DAPI (Donin Chemical Laboratory, cat. no. D523) was added and pipetted, and the rate of hCD45 positivity was measured using a flow cytometer (Novocyte 3000, ACEA Biosciences, Inc.) through a tube with a 35-μm cell strainer.

**H&E staining in the MOLM-13 xenograft model.**

The presence of tumor cells in the MOLM‑13 transplantation model treated with DS-1594a·HCl was histopathologically confirmed by H&E staining in the femoral bone marrow of NSG mice. For staining, the femurs from NSG mice were fixed with 10% (v/v) neutral buffered formalin for approximately 48 h and then demineralized with EDTA for 5–6 days. The femurs were longitudinally paraffin-embedded to include the knee joint after decalcification. The paraffin‑embedded tissues were sectioned, and H&E staining was performed according to conventional methods.^15^ NanoZoomer 2.0-HT (Hamamatsu Photonics) was used to create virtual slides of the H&E-stained specimens. Histopathological evaluation was performed by observing the stained specimens with light microscopy and a virtual slide-viewing software (NDP.view2, Hamamatsu Photonics).

**Repeated-dose hematological toxicity study in mice.**

DS-1594a·succinate suspended in 0.5% methylcellulose solution was orally administered by gavage to Crl:CD1(ICR) mice (10 mice/group) at doses of 0 (vehicle control), 30, 100, and 300 mg/kg (the doses are expressed as DS-1594a, the free form of DS-1594a·succinate) for 28 consecutive days. Five animals receiving doses of 0 and 300 mg/kg were used to evaluate the reversibility of toxicity during a 28-day recovery period. As much blood as possible was drawn from the caudal vena cava with a syringe containing heparin sodium from animals anesthetized for necropsy, and 0.3 mL of blood was treated with an anticoagulant (EDTA-2K) for measurement with an XT-2000iV fully automated hematology analyzer (Sysmex Corporation). This study was approved by the IACUC (Approval No. IACUC315-895) and was performed in accordance with the animal welfare bylaws of Shin Nippon Biomedical Laboratories, Ltd., which is accredited by AAALAC International.

**RNA purification and RT‒qPCR.**

For murine MA9 cells, total RNA was extracted after 4 days of treatment with DMSO or DS-1594a·HCl. For patient-derived cells, total RNA was extracted after 7 days of treatment with DMSO, DS-1594a·HCl, DS-1594a·succinate, or Ara‑C. For animal bone marrow cells, RNA was extracted 1 day after the last treatment with vehicle or DS-1594a·HCl or DS-1594a·succinate. All RNA was extracted using an illustra RNAspin Mini RNA Isolation Kit (GE Healthcare) or an RNeasy^®^ Mini Kit (QIAGEN) per the manufacturer’s instructions and measured using a NanoDrop 8000 ultraviolet-visible spectrophotometer (Thermo Fisher Scientific). The mRNA expression levels of individual genes (*Meis1*, *Hoxa9*, *Mef2c*, *Pbx3*, and *Actb* for murine MA9 cells; *MEIS1*, *HOXA9*, *ACTB, and GAPDH* for patient-derived cells; *MEIS1*, *HOXA9, MEF2C*, *PBX3,* and *ACTB* for the MOLM-13 xenograft model; and *MEIS1*, *MEF2C*, *CDK6,* and *GAPDH* for the *MLL1-r* ALL-PDX model) were quantified by RT‒qPCR using TaqMan™ Fast Virus 1-Step Master Mix (Thermo Fisher Scientific Inc.), a THUNDERBIRD Probe One-step qRT‒PCR Kit (TOYOBO CO., LTD., cat. no. QRZ-101), or TaqMan™ Universal PCR Master Mix (Thermo Fisher Scientific, cat. no. 4364340) and measured with a ViiA7 real-time PCR system (Thermo Fisher Scientific, Inc.). All TaqMan probes used to detect the respective mRNA transcripts were purchased from Thermo Fisher Scientific, Inc.

**Probes used for real-time PCR.**

The following TaqMan probes were used to quantify the expression of individual genes:

For RNA from murine MA9 cells:

*Meis1*: Mm00487664_m1, *Hoxa9*: Mm00439364_m1, *Mef2c*: Mm01340842_m1, *Pbx3*: Mm00479413_m1, *Actb*: Mm02619580_g1, *Kit*: Mm00445212_m1, *Itgam* (*Cd11b*): Mm00434455_m1, *Ly6g*: Mm.PT.58.30498043.

For RNA from cell lines, patient-derived cells, or animal bone marrow cells:

*MEIS1*: Hs00180020_m1 or Hs01017441_m1, *HOXA9*: Hs00365956_m1, *HOXA7*: Hs00600844_m1, *RUNX1*: Hs.PT.58.24461868, *MYB*: Hs.PT.58.264008, *JMJD1C*: Hs.PT.58.892852, *MEF2C*: Hs00231149_ml, *PBX3*: Hs00608415_ml, *ACTB*: Hs01060665_g1, *GAPDH*: Hs02786624_g1, *CD34*; Hs.PT.56a.24708916, *ITGAM* (*CD11b*); Hs.PT.58.40141028,

For RNA from animal bone marrow cells:

*MEIS1*: Hs01017441_m1, *MEF2C*: Hs00231149_ml, *CDK6*: Hs01026371_m1, *GAPDH*: Hs02758991_g1

***Supplemental References***

1. Huang J, Gurung B, Wan B, Wan K, Hua X, Lei M. The same pocket in menin binds both MLL and JUND but has opposite effects on transcription. Nature. 2012;482(7386):542-546.

2. Kabsch W. Xds. Acta Crystallogr D Biol Crystallogr. 2010;66(Pt 2):125-132.

3. Evans PR, Murshudov GN. How good are my data and what is the resolution? Acta Crystallogr D Biol Crystallogr. 2013;69(Pt 7):1204-1214.

4. McCoy AJ, Grosse-Kunstleve RW, Adams PD, Winn MD, Storoni LC, Read RJ. Phaser crystallographic software. J Appl Crystallogr. 2007;40(Pt 4):658-674.

5. Murshudov GN, Skubák P, Lebedev AA, et al. REFMAC5 for the refinement of macromolecular crystal structures. Acta Crystallogr D Biol Crystallogr. 2011;67(Pt 4):355-367.

6. Emsley P, Cowtan K. Coot: model-building tools for molecular graphics. Acta Crystallogr D Biol Crystallogr. 2004;60(Pt 12 Pt 1):2126-2132.

7. The PyMOL Molecular Graphics System, Version 1.7.2.1 Schrödinger, LLC. <https://pymol.org/2/>. Accessed 7 February 2022.

8. Subramanian A, Tamayo P, Mootha VK, et al. Gene set enrichment analysis: a knowledge-based approach for interpreting genome-wide expression profiles. Proc Natl Acad Sci U S A. 2005;102(43):15545-15550.

9. Bernt KM, Zhu N, Sinha AU, et al. MLL-rearranged leukemia is dependent on aberrant H3K79 methylation by DOT1L. Cancer Cell. 2011;20(1):66-78.

10. Gene Set: HESS_TARGETS_OF_HOXA9_AND_MEIS1_UP. <https://www.gsea->msigdb.org/gsea/msigdb/cards/HESS_TARGETS_OF_HOXA9_AND_MEIS1_UP. Accessed February 7, 2022.

11. Gene Set: HESS_TARGETS_OF_HOXA9_AND_MEIS1_DN. <http://www.gsea->msigdb.org/gsea/msigdb/cards/HESS_TARGETS_OF_HOXA9_AND_MEIS1_DN. Accessed February 7, 2022.

12. Ng SWK, Mitchell A, Kennedy JA, et al. A 17-gene stemness score for rapid determination of risk in acute leukaemia. Nature. 2016;540(7633):433-437.

13. Elsayed AH, Rafiee R, Cao X, et al. A six-gene leukemic stem cell score identifies high risk pediatric acute myeloid leukemia. Leukemia. 2020;34(3):735-745.

14. Robinson MD, McCarthy DJ, Smyth GK. edgeR: a Bioconductor package for differential expression analysis of digital gene expression data. Bioinformatics. 2010;26(1):139-140.

15. Fischer AH, Jacobson KA, Rose J, Zeller R. Hematoxylin and eosin staining of tissue and cell sections. CSH Protoc. 2008;2008:pdb.prot4986.
