# Supplementary figures and images for: A novel Menin-MLL1 inhibitor, DS-1594a, prevents the progression of acute leukemia with rearranged MLL1 or mutated NPM1
Source: Cancer Cell Int. 2023 Feb 25;23:36. doi: 10.1186/s12935-023-02877-y (PMC9960487; doi:10.1186/s12935-023-02877-y)

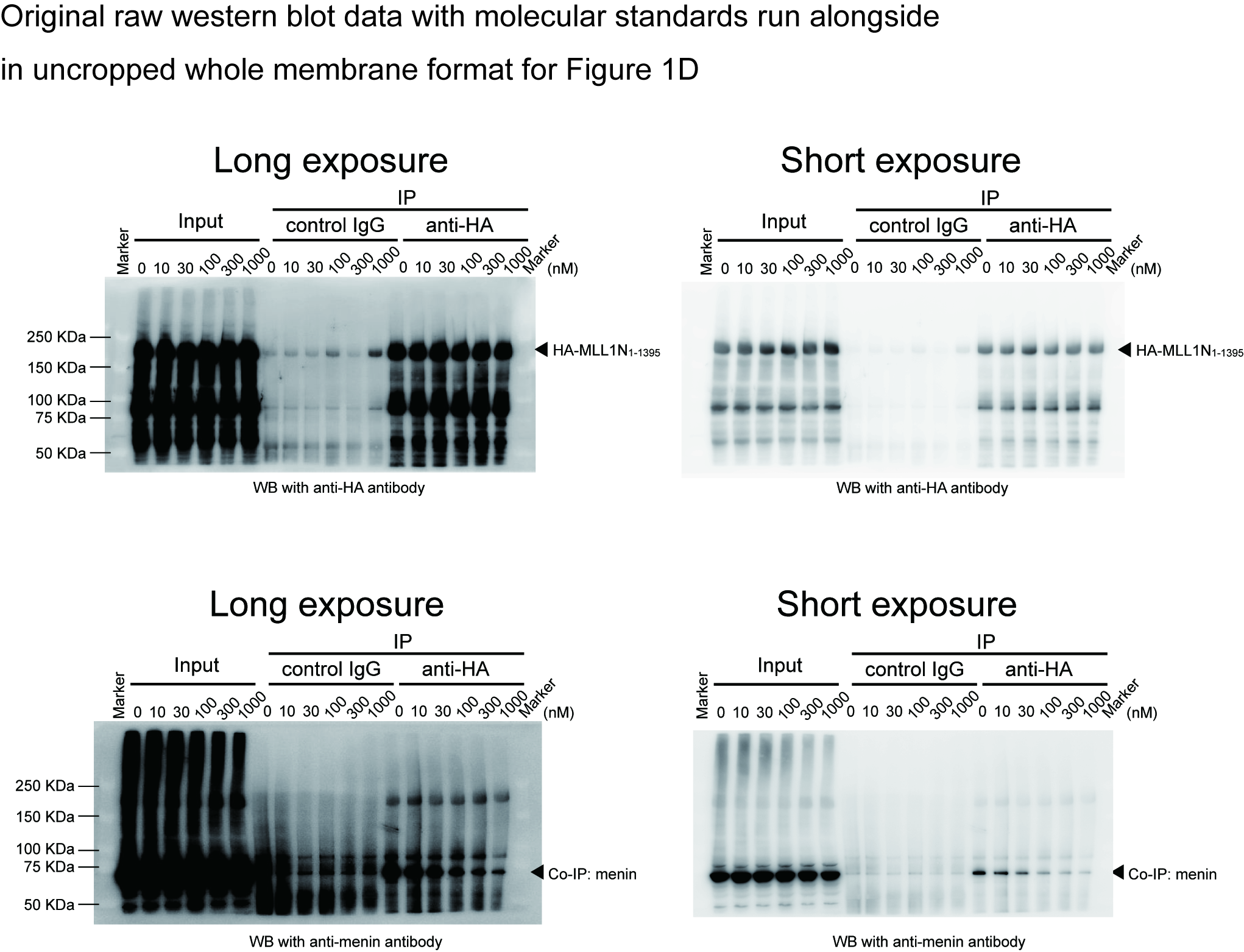

Supplement: Supplementary file 2 — Additional file 2. Original raw WB data for Fig. 1D. [file 12935_2023_2877_MOESM2_ESM.tif]
